# Sharing power in global health research: an ethical toolkit for designing priority-setting processes that meaningfully include communities

**DOI:** 10.1186/s12939-021-01453-y

**Published:** 2021-05-25

**Authors:** Bridget Pratt

**Affiliations:** Centre for Health Equity, School of Population and Global Health, University of Melbourne, 207 Bouverie St, Carlton, Victoria 3053 Australia

**Keywords:** Ethics, Power, Social justice, Health research, Engagement, Involvement, Community

## Abstract

To promote social justice and equity, global health research should meaningfully engage communities throughout projects: from setting agendas onwards. But communities, especially those that are considered disadvantaged or marginalised, rarely have a say in the priorities of the research projects that aim to help them. So far, there remains limited ethical guidance and resources on how to share power with communities in health research priority-setting. This paper presents an *“ethical toolkit”* for academic researchers and their community partners to use to design priority-setting processes that meaningfully include the communities impacted by their projects. An empirical reflective equilibrium approach was employed to develop the toolkit. Conceptual work articulated ethical considerations related to sharing power in g0l0o0bal health research priority-setting, developed guidance on how to address them, and created an initial version of the toolkit. Empirical work (51 in-depth interviews, 1 focus group, 2 case studies in India and the Philippines) conducted in 2018 and 2019 then tested those findings against information from global health research practice. The final ethical toolkit is a reflective project planning aid. It consists of 4 worksheets (Worksheet 1- Selecting Partners; Worksheet 2- Deciding to Partner; Worksheet 3- Deciding to Engage with the Wider Community; Worksheet 4- Designing Priority-setting) and a Companion Document detailing how to use them. Reflecting on and discussing the questions in Worksheets 1 to 4 before priority-setting will help deliver priority-setting processes that share power with communities and projects with research topics and questions that more accurately reflect their healthcare and system needs.

**Supplementary Information:**

The online version contains supplementary material available at 10.1186/s12939-021-01453-y.

## Introduction

Social justice is a foundational moral value of health research, policy, and practice [1–3]. Three core aspects of social justice are: (1) addressing the health needs of those considered disadvantaged or marginalised, (2) supporting their participation in decision-making, and (3) combatting epistemic injustice by drawing out and listening to the experiences and perspectives of those whose voices have been marginalised, silenced, devalued, inferiorised, and/or erased [3–5]. Disadvantage means experiencing sizeable deficits on multiple dimensions of well-being [4, 5]. Dimensions of well-being include health; respect; personal security; reasoning; control over one’s environment; sense, imagination, and thought; and attachment [4–6]. Mechanisms that create and sustain disadvantage include: 1) forms of oppression and subordination such as colonialism, racism, gender bias, and stigmatization of members of groups and 2) concentrations of power, resources, and privileged social standing that result in the structuring of social, economic, and political arrangements to benefit dominant groups and reinforce existing inequalities [4]. They make it much harder for certain groups and individuals to achieve well-being. Being socially marginalised means that individuals and groups experience disadvantage due to unequal treatment by social institutions and norms. They are excluded from participating fully in the economic, social, and political life of the society in which they live [7].

To promote social justice and equity, health research should focus on the health needs of those considered disadvantaged or marginalised by social institutions and norms [1, 3, 4]. It is essential that the health concerns and knowledge of marginalised communities are visible in health research topics and questions. Health research should also meaningfully engage such communities throughout research projects: from setting agendas and research design to determining how data is collected and analysed and what happens with findings and outputs [8, 9]. This means community members are able to have a say and influence (amongst other things) the priority-setting process and its outputs. Their engagement in priority-setting is a key means for setting research topics and questions that matter to them [9]. Where research projects are less relevant to communities, they are much less likely to generate evidence that will improve public health and healthcare systems for them [9].

Yet communities, especially those considered disadvantaged or marginalised, rarely have a say in the agendas of the very global health research projects that aim to help them. These agendas are largely set by researchers and funders, often from high-income countries. Even where community engagement occurs, without attention to power dynamics, it can lead to tokenism: presence without voice and voice without influence. Voices are excluded from priority-setting, particularly those already disadvantaged or marginalised by their societies’ institutions and norms. Existing evidence confirms that, for example, being female, being poor, having little formal education, living with a disability, and/or belonging to certain ethnic groups means community members are listened to less or not at all during health priority-setting [10]. It is, therefore, very important to carefully design global health research priority-setting processes to share power with communities.

What is needed to ensure marginalised communities’ voices are raised and heard in agenda-setting for global health research projects? So far, there remains limited ethical guidance on this matter. While existing literature explores the concept of participation in decision-making in contexts of power disparities, spanning disciplines like political philosophy, ethics, development studies, health policy, and community-based participatory research [11–24], that body of work largely does not consider the participation of marginalised communities in the context of health research priority-setting [25]. Existing ethical guidance on community engagement in global health research (e.g. CIOMS *International ethical guidelines for health-related research involving humans* (2016), UNAIDS *Good participatory practice guidelines for HIV prevention trials* (2011), NIAID’s *Recommendations for community involvement in National Institute of Allergy and Infectious Diseases HIV/AIDS clinical trial research* (2009)) generally does not discuss how power imbalances should be dealt with or priority-setting [26]. Unsurprisingly then, tools and resources have not been created to help researchers and their partners share power with communities in their priority-setting practice.

To address this gap in guidance and resources, a three-year program of empirical ethics research was undertaken. It aimed to characterise the ethical considerations related to sharing power that should be taken into account when engaging communities in priority-setting for global health research projects and to develop guidance on how to address them. It would further create a resource—*an “ethical toolkit”*—for researchers and their partners to use in their practice that incorporated the findings of that research. The toolkit would help researchers and their partners design priority-setting processes that meaningfully include the communities impacted by their projects.

In this paper, the development of the ethical toolkit is described and the final version of the toolkit is presented for the first time. The toolkit is a reflective project planning aid to employ before priority-setting is undertaken for global health research projects. It consists of four worksheets (to be completed collectively by the research team) and a companion document detailing how to use them.

## Developing the toolkit

A *reflective equilibrium* approach [27, 28] was employed to develop the ethical toolkit. Traditionally, reflective equilibrium entails working back and forth between theoretical considerations (intuitions, moral principles, theories) and empirical facts/information reported in the literature, testing existing theoretical considerations against new ones or newly reported empirical knowledge, revising, and refining until coherence is achieved [29]. Reflective equilibrium as a methodology in empirical work, despite using different tools and a wider set of judgements, is continuous with reflective equilibrium in conceptual work. In *empirical ethics research*, reflective equilibrium entails testing theoretical considerations against information gathered from practice—namely, the considered judgements of people who are involved the practice-under-study—using empirical methods (e.g. interviews, surveys, case studies) [27, 30]. It is a standard and rigorous approach in ethics research and was selected because it comprises a robust methodology for developing ethical guidance that is informed by both theory and practice [30]. The considered judgements used in this project were those of people involved in global health research: academic researchers, ethicists, community engagement practitioners, health provider research partners, community organisation research partners, study participants, and people with lived experience and members of the public who had been engaged in health research.

### Conceptual work

To identify theoretical considerations, six key bodies of literature that discuss participation in contexts of power disparities were analysed for components of engagement and sites of power. The bodies of literature included development studies, political philosophy, ethics, health priority-setting, public deliberation, and community-based participatory research. Four components of engagement were identified—who initiates, for what purpose, who participates, and how they participate^1^ and each had several associated sites of power [25]. Sites of power are features of priority-setting (e.g. ground rules, facilitation) that affect who shapes priority-setting processes, who participates, and who is heard in them. How sites of power might affect engagement in global health research priority-setting was explored. Ethical considerations related to sharing power at each site were then characterised for the global health research priority-setting context and guidance on how to address them was developed [25]. An initial version of the toolkit was created, with its content reflecting the ethical considerations (and their associated sites of power) and guidance generated by the conceptual work [25].

### Empirical work

#### Data collection

Components of engagement, sites of power, and ethical considerations identified by the conceptual work were then tested against and informed by the experiences and perspectives of researchers, ethicists, community engagement practitioners, community-based organisation staff, and people with lived experience and members of the public who had been engaged in health research. In 2018 and 2019, 51 in-depth interviews and 1 focus group were performed as well as 2 case studies of global health research priority-setting processes where communities were involved as partners. The focus group was conducted with 9 ethicists in the UK with expertise in global health ethics.

Those recruited for in-depth interview came primarily from Australia, the UK, Europe, and Africa and, to a lesser extent, from North America, Latin America, and Southeast Asia. Sampling was initially purposive; potential participants with experience undertaking engagement with disadvantaged groups in health research, with expertise in community engagement ethics, and with lived experience who had been involved in health research were identified through BP’s existing networks. Snowball sampling was then used to identify additional interviewees. In total, 36 women and 15 men were interviewed. Sixteen interviews and the focus group discussion were conducted in person in Australia and the UK; 35 interviews were performed over Skype or the phone. Interviews were approximately 30 to 85 min’ duration.

Following the technique of thick description, interview questions were open-ended [32] and attempted to draw out interviewees’ experience with and perspectives on power-sharing in health research priority-setting. Interviewees were first asked to describe either their experience in community engagement and health research, especially with marginalised groups and communities, or what roles they had been engaged in as part of health research projects. Researchers, ethicists, community-based organisation staff, and community engagement practitioners were then asked what is important to balance or share power when community members participate in health research priority-setting for projects. People with lived experience and members of the public who had been engaged in health research were asked: What’s necessary for people like yourself to meaningfully participate in health research? When people like you are involved in health research priority-setting, what is important to share power with them? Questions were kept general to draw out interviewees’ ideas about power-sharing and then probes were used to further explore their ideas’ meaning. Interviews and focus group discussions were transcribed verbatim and thematic analysis was undertaken by two coders (BP and NE) in the following five phases: initial coding framework creation, coding, inter-coder reliability and agreement assessment, coding framework modification, and final coding of entire dataset [33, 34]. Some of this data (excluding the 22 interviews with people with lived experience and members of the public) is published [35, 36].

The two retrospective case studies were of priority-setting in global health research projects in India and the Philippines respectively (see Table 1). For the case study of the Participation for Local Action (PLA) project in India, 14 semi-structured in-depth interviews were conducted in Bangalore and the BR Hills in December 2018 and April 2019 with researchers (four interviews), members of their Indigenous community organisation partner (five interviews), and field investigators from the Indigenous community (five interviews). Interviews with community organisation members and field investigators were performed in Kannada with the assistance of a research assistant (NSN). A meeting between the community organisation partner and researchers was observed by BP and NSN in April 2019 to supplement the interview data. This meeting was not about the PLA project but a subsequent project being conducted by the researchers and the community organisation. Direct observation notes were taken regarding the dynamics between the researchers and members of the Indigenous community organisation partner: namely, who led the meeting, where people physically sat, who spoke, who was silent or listening, what questions were asked by members of the Indigenous community organisation to the researchers, whether members of the Indigenous community organisation challenged or disagreed with things the researchers said, who made decisions, whose voices were reflected in the decisions that were made, and whether consensus was reached.

**Table 1:** Case studies

**Participation for Local Action project (India)** The Participation for Local Action (PLA) project ran from January 2015 to September 2016. Its research team consisted of seven members: a principal investigator from a local NGO, one co-investigator from the Institute of Public Health in Bangalore, one co-investigator from the Zilla Budakattu Girijana Abhivrudhhi Sangha [district Sangha], two co-investigators from other Indian research institutions, one co-investigator from a Belgian university, and two co-investigators from the Government of Karnataka. The PLA project was undertaken in partnership with the district Sangha, an Indigenous community development organisation. The district Sangha is comprised of 21 members [district Sangha leaders] and represents all Soliga living in Chamarajanagar district. The Soliga are an Indigenous population who have lived in the BR Hills region of Southern Karnataka for centuries. The district is divided into four sub-districts: Chamarajanagar, Gundlupet, Kollegal and Yelandur. Sangha organisations also exist at sub-district and village levels. The PLA project was a participatory action research project that aimed to improve maternal and child health in the Soliga community. The project essentially comprised a priority-setting process. Introductory meetings were first conducted in 2015 between the researchers and district Sangha leaders. Once the district Sangha decided to participate, it recruited ten field investigators (five men and five women from the Soliga community) and a field supervisor to visit the district’s 148 Soliga villages and collect data about the problems that households faced in accessing maternal and child health services. Data were jointly analysed by three district Sangha leaders and one of the researchers, and six core themes were identified. In June 2016, a deliberative workshop was held and attended by 60 Sangha leaders (from district, sub-district, and village levels) to reflect on the six themes, to prioritise amongst them, and to develop community-led solutions to address them. A health navigator intervention (a mobile helpline for health queries) was selected for implementation. **Women with Disabilities taking Action on REproductive and sexual health project (Philippines)** The Women with Disabilities taking Action on REproductive and sexual health (W-DARE) project ran from 2013 to 2015. It comprised an interdisciplinary partnership between researchers, health service providers, and disabled persons organisations that steered the engagement of women living with disability in Quezon City and Ligao City in the Philippines. It was conducted in partnership by the University of Melbourne (Australia); De La Salle University (Philippines); community partners WOWLEAP, PARE, and Likhaan Center for Women’s Health; and the Center for Women’s Studies at the University of the Philippines. The W-DARE project was a participatory action research project that aimed to improve the sexual and reproductive health of women with disability in the Philippines. The project supported disabled persons organisations and women living with disability to identify and prioritise issues related to accessing reproductive and sexual health services and to develop solutions/interventions to address them. The W-DARE project essentially comprised a five-stage priority-setting process: 1. Conceptualising- Setting aims and methods to generate priorities 2. Planning- Designing data collection tools and determining how to implement them 3. Data collection and analysis- Identifying main health problems through in-depth interviews and focus groups with women living with disability, their families, and service providers 4. Deciding which problem(s) to prioritise 5. Identifying interventions and prioritising which to take forward Women living with disability participated in stages two through five as part of the research team. Women living with disability from Quezon City and Ligao City also participated in stage 3 as study participants. Through this process, a set of interventions was selected that included demand-side interventions, supply-side interventions, interventions to develop an enabling local environment, and enabling society interventions that were then tested.	

For the case study of the Women with Disabilities taking Action on REproductive and sexual health (W-DARE) project in the Philippines, 18 semi-structured interviews were conducted in Quezon City in September 2019 with researchers, their community and government partners, and study participants. Interviewees spanned all W-DARE partners: the University of Melbourne, De La Salle University, University of the Philippines, Women with Disabilities LEAP to Social and Economic Progress, Inc. (WOWLEAP), PARE, Likhaan Center for Women’s Health, and Ligao City government. WOWLEAP and PARE are disabled persons organisations; Likaan is a community-based health service provider. Eight interviewees had lived experience of physical disability (mobility, hearing, visual). Eight interviews were performed in Tagalog with the assistance of a research assistant (MP). In both case studies, project-related documents were collected. Interviews were transcribed verbatim and thematic analysis was undertaken by two coders (BP and NE for the PLA project case; NE and MW for the W-DARE project case) as above. Interview transcripts, direct observation notes, and project-related documents were included in that thematic analysis.

#### Data analysis

Data was analysed separately for the interviews with researchers, ethicists, and community engagement practitioners; the interviews with people with lived experience and members of the public; and each case study. However, across the empirical studies informing the toolkit, three main themes (or components of engagement) were identified through thematic analysis: context, process, and aftermath. Components of engagement identified by the conceptual analysis— who initiates, for what purpose, who participates, how they participate—fell within the process theme. The context theme encompasses features of the research setting, research team, and community of focus that can either facilitate or obstruct power-sharing during priority-setting and the formation of partnerships prior to priority-setting. Subcategories under this theme included: selecting academic partners, selecting community partners, deciding to engage with researchers, deciding to engage with community partners, and deciding to engage with the wider community. Selecting academic or community partners encompasses features of academic or community partners that are important to sharing power with each other and with communities, especially those who are considered disadvantaged or marginalized, in global health research. Deciding to engage categories encompass relational and environmental factors that can either facilitate or obstruct power-sharing in global health research priority-setting and the processes by which academic and community partners decide to engage with each other and with the wider community.

The temporal themes of process and aftermath encompass the sites of power that exist during and after global health research priority-setting. Additional sites of power (coded as subcategories under the process theme) not identified by the conceptual work were described by the empirical work, including listening, ground rules, space, and accountability (Table 2). The empirical work also identified sites of power that were captured by the conceptual analysis, including leadership, level of participation, stage of participation, representation, and mass (Table 2).

**Table 2:** Sites of power

Site of power	Definition
Leadership	Who takes the lead on the key aspects of research priority-setting: planning, implementing, ensuring outputs are fed back and used.
Scope	What issues can be brought into the priority-setting space and what issues are not allowed; What information is presented or shared with participants at the start of the priority-setting process.
Empowerment	What goals are set for the priority-setting process: instrumental and/or empowerment. Power-sharing is promoted where engagement processes aim to *empower* community partners and members as researchers and to be involved in research priority-setting respectively.
Stage of participation	When community partners and members participate in the health research priority-setting process.
Level of participation	The mode of participation assumed by community partners and members during health research priority-setting. *Decision-making* means being responsible for making key choices in priority-setting, e.g. process planning choices, research topic selection, research question selection. *Consulting* means giving input but having no assurance that it will be used by those who decide.
Diversity within the community	The range of people engaged from the community.
Representation	Being present or represented during health research priority-setting.
Mass	Numbers of community partners and members of the wider community relative to academic researchers.
Space	The physical setting in which health research priority-setting is undertaken.
Ground Rules	Rules under which health research priority-setting is undertaken. They specify who can and cannot be present, who can speak, when individuals can speak, what roles individuals play in the process, how different individuals’ views are used, and how a decision or closure is reached.
Facilitation	Whether facilitators and chairs of discussions and meetings ensure everyone (researchers, community partners, community members) has an equal opportunity to speak and draw out quieter voices.
Listening	Whether researchers engage in dialogue (ask questions and for clarifications) about what community partners and members say *and* document what they have said.
Being heard	Whether the views of community partners and members are taken on board and the information they provide is acted upon. Ideally, a joint product is created with inputs from researchers and those engaged.
Resources and compensation	Whether community partners are employed as members of the research team and community members are paid for time worked, rather than covering their expenses.
Unintended harms	Possible harms that could eventuate for community members from being part of a priority-setting process.
Accountability	Responsibilities of researchers, research institutions, and community partners after priority-setting.

### Reflective equilibrium

By comparing the outputs of the conceptual and empirical work to one another, the toolkit was revised twice. Where additional components of engagement (i.e. context, aftermath) and sites of power were identified by the empirical work, conceptual work, drawing on power-sharing strategies described in the empirical work, was then performed to derive ethical considerations relevant to them. Where previously identified components of engagement and sites of power were identified, ethical considerations from the conceptual work were examined in light of empirical data and refined.

The resultant final toolkit reflects conceptual coherence by adapting conceptual analysis in light of empirical evidence, and, where appropriate, using social and ethical theory to challenge the intuitions evidenced in the empirical data [37]. The context theme subcategories ‘selecting academic partners’ and ‘selecting community partners’ largely generated the content of Worksheet 1: Selecting partners. The context theme subcategories around ‘deciding to engage’ largely generated the content of Worksheet 2: Deciding to Partner and Worksheet 3: Deciding to Engage with the Wider Community. The sites of power identified through the conceptual and empirical work generated the content of Worksheets 4A and 4B: Designing Priority-setting. The WDARE case study gave particular insight into which sites of power are relevant when priority-setting occurs within the research team. The interviews and PLA case study gave insight into which sites of power are relevant when priority-setting occurs with the broader community.

The second version of the toolkit is presented in Pratt [38]. The third and final version is described in this paper for the first time. Unlike the second version, it incorporates the findings of the two case studies and the findings of 22 interviews with people with lived experience and members of the public who had been engaged in health research. *These case studies and interviews were performed to ensure the toolkit was better informed by the views of community organisations and community members.* How the third version of the toolkit differs from the second version is discussed below.

## The final Toolkit

### Version 3 of the toolkit

**Fig. 1:**
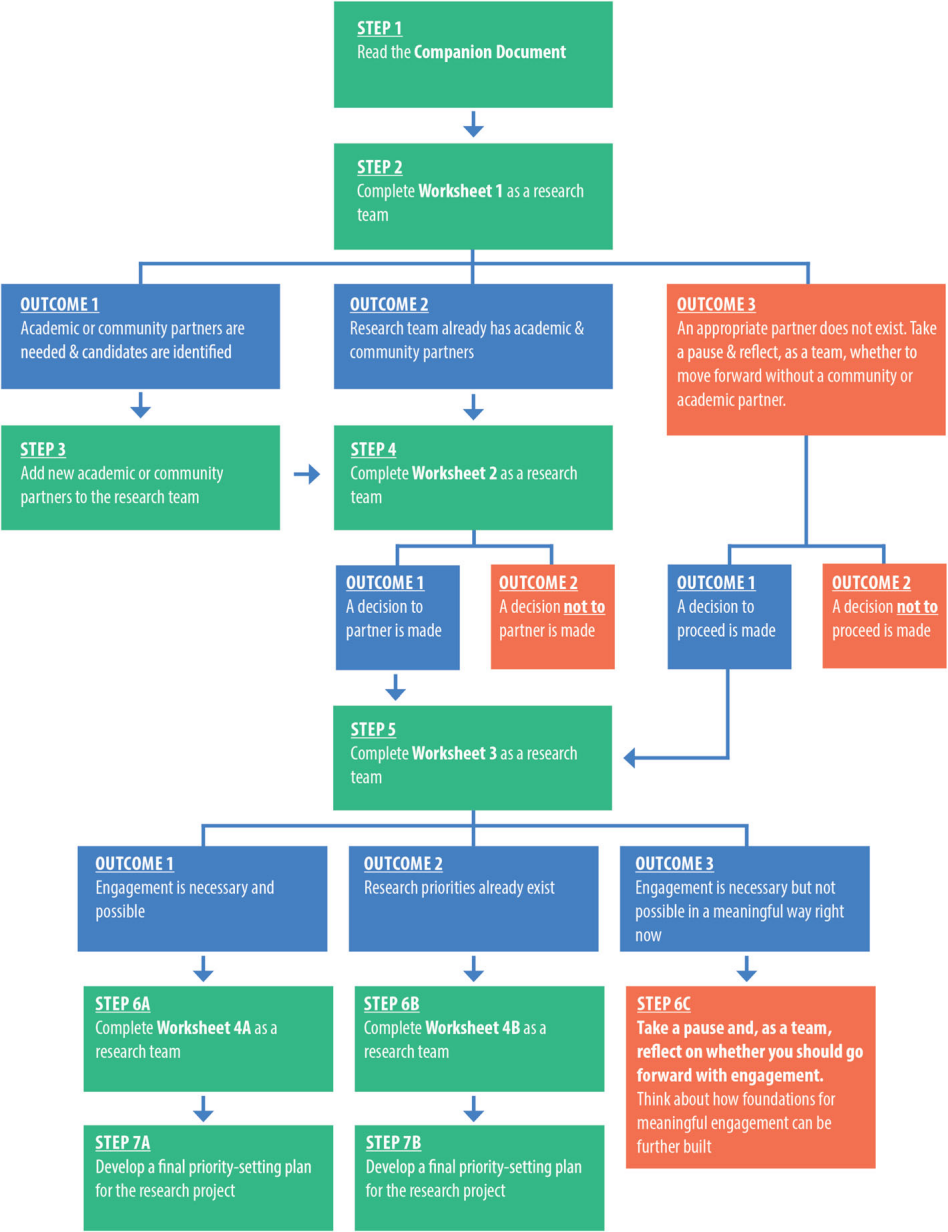
Step by step guide on how to use the toolkit

The final version of the toolkit for *Sharing Power with Communities in Priority-Setting for Health Research Projects* is a set of four worksheets and a companion document (Supplemental Files 1, 2, 3, 4, 5 and 6). The toolkit is a reflective project planning aid for use *before* undertaking priority-setting for a global health research project. It should be used, as described in Fig. 1, to develop and inform a final priority-setting plan. Academic researchers and their community partners (e.g. community organisations, disabled persons organisations, health care providers, policymakers) should use the toolkit together. This means completing Worksheets 1 to 4 as a team.

The *Companion Document* provides toolkit users with information about the toolkit (e.g., its purpose, how it was developed, who should use it and how) and with guidance on how to understand worksheet questions and why they are important. It should be read first. *Worksheet 1* helps research teams think about and collectively determine whether they can be strengthened by adding an (or additional) academic or community partner(s). It has two questions for reflection and discussion (Table 3) and provides further guidance on what features to look for to select prospective academic partner(s) who can help improve the condition of a given community and prospective community partner(s) that represent and can access a community that is considered disadvantaged or marginalised in its diversity.

**Table 3:** Summary of toolkit questions for reflection and discussion by worksheet

	Questions
**Worksheet 1**	**1. Partners** For communities: Can your prospective academic partner(s) help improve the condition of the community you are part of or that your organisation serves? For academic researchers: Does your prospective community partner(s) represent and can it/they access a community that is considered disadvantaged or marginalised in its diversity? **2. Framing** How will you frame the priority-setting process to the community/academic partner(s) being approached?
**Worksheet 2**	**1. Building Foundations** How will relationships between partners be built or made stronger? How will community partners be supported to participate in priority-setting? **2. Barriers** What barriers still might exist to sharing power between partners?
**Worksheet 3**	**1. Existing Priorities** Have the community’s health research priorities, including those of the disadvantaged, less influential, lower status, and/or marginalised, already been voiced? **2. Building Foundations** How will relationships between the research team and the wider community be built or made stronger before priority-setting starts? How will community members be supported to participate in priority-setting? **3. Barriers** What barriers to meaningful engagement with the wider community might still exist?
**Worksheet 4A**	**1. Leadership** Who will lead the health research priority-setting process? **2. Scope** Will research topics be solicited relating to *all* health problems experienced by community members? **3. Empowerment** Will community partners be empowered as researchers during priority-setting? Will community members’ capacity to participate in research priority-setting be strengthened? **4. Stage of Participation** What stage(s) of the priority-setting process do community partners want to be involved in? What stage(s) of the priority-setting process will community members be involved in? Is this acceptable to them?* **5. Level of Participation** Will community members be involved as collaborators (decision-makers) and/or consultants? Is this acceptable to them?* **6. Diversity within the community** 6a. Which community roles will you engage during priority-setting and for what reasons? 6b. List which of the roles identified in Q6a correspond to greater or lesser influence and status within the community. 6c. Who are considered disadvantaged, less influential, lower status, or marginalised within these roles? 6d. Which of those groups or stakeholders in Q6c will you engage and for what reasons? 6e. Is it fair to bring these community members into the same decision-making space? **7. Representation** Which organisations or individuals will represent the roles listed in Q6a in priority-setting? Do these representatives encompass those considered disadvantaged, less influential, lower status, and/or marginalised within each role, as identified in Question 6c? **8. Mass** Will the number of community partner staff and community members be greater or equal to academic partner staff in consultations and deliberations during priority-setting? Will the number of representatives of lower status community roles (identified in Q6b) be sufficient at consultations and deliberations during priority-setting? **9. Space** Where will you hold the priority-setting process for your research project? **10. Ground Rules** Will community partners and members be involved in developing and approving the ground rules for the priority-setting process? If not, what are your reasons? What ground rules will you include to ensure stakeholders identified in Question 6d aren’t silenced during priority-setting? **11. Facilitation** Will you have a locally-based person facilitate consultations and deliberations during priority-setting? If not, what are your reasons? How will the facilitation method/approach give participants an equal opportunity to speak at consultations and deliberations during priority-setting? How will the facilitation method/approach make community partners and members feel comfortable sharing relevant, personal stories about their community’s health concerns at consultations and deliberations? **12. Listening** How will the research team ensure community partners’ and members’ ideas are listened to during consultations and deliberations? **13. Being Heard** Will the voices of community members, especially those considered disadvantaged, less influential, lower status, and/or marginalised, have equal or greater weight than other participants’ voices when setting research priorities? If not, what are your reasons? **14. Resources and Compensation** How will community partners and communities be compensated for participating in priority-setting? Will community partners have control over any project resources? Will full information about the research project’s budget be disclosed to community partners? **15. Unintended harms** What harms to community partners or members do you think might result from the priority-setting process? **16. Accountability** Will the research team act upon the final research topic and questions? How will the final research topic and questions be fed back to field investigators and community members, including those considered disadvantaged, less influential, lower status, and/or marginalised, after priority-setting? How are the research team and community members going to evaluate community members’ engagement in the priority-setting process?

*To ascertain once specific community members are invited to participate

*Worksheet 2* helps research teams reflect on and collectively determine whether they can share power within their partnership. Two types of foundations—relational and environmental—were identified as essential to have in place before power sharing can occur between academic and community partners. The worksheet’s two questions ask research teams to reflect on whether these foundations are in place in their partnership (Table 3). If they are not, Worksheet 2 offers guidance on how to build them.

Once the research team and partnership is finalised, *Worksheet 3* helps its members reflect on and collectively determine whether wider community engagement is necessary in priority-setting and, if so, whether it can be meaningfully done with members of a given community. In some settings, previous studies may have captured community members’ views and ideas, including those of the disadvantaged or marginalised, that speak to or explicitly articulate their health research priorities. Where community health research priorities have already been articulated, it may not be necessary to undertake community engagement activities in priority-setting for new projects. Doing so, in fact, might comprise a poor use of resources that could be better spent elsewhere. Where community research priorities do not exist, having relational and environment foundations in place with the wider community are again essential for power-sharing to occur. The worksheet’s questions ask research teams to reflect on whether community research priorities already exist and, if not, to consider whether those foundations are in place between the research team and the wider community (Table 3). If they are not, Worksheet 3 offers guidance on how to build them.

Where meaningful engagement is necessary and possible, *Worksheet 4A* then helps research teams design the priority-setting process for a given global health research project. Reflecting on and collectively answering Worksheet 4A questions (Table 3) will promote the design of priority-setting processes where power is more evenly shared with communities. Worksheet questions correspond to the 16 sites of power in the global health research priority-setting process that were identified by the conceptual and empirical work (Table 2). Where meaningful engagement is not necessary or possible, *Worksheet 4B* then helps research teams undertake a priority-setting process for a global health research project where power is shared between academic and community partners. It has questions for reflection and discussion that correspond to 12 sites of power in the research priority-setting process. Those questions are similar to those found in Worksheet 4A, but they are framed in the context of sharing power between academic and community partners (rather than the research team and wider community).

In addition to providing questions for research teams to reflect on and discuss, the worksheets suggest next steps for research teams to take based on their answers. Table 4 provides an example.

**Table 4:** Example of guidance provided in Worksheets

**Worksheet 4A, Question 5** Will community members be involved as collaborators (decision-makers) and/or consultants? Is this acceptable to them? **Suggested next steps:** - If your answer to Question 5 is that community members will be involved as collaborators, consider whether and how research priorities can be set through a deliberative process with community members that yields a collective decision. Deliberative processes are governed by norms of equality, symmetry, and non-coercion and generate consensus. All participants can voice their ideas for research priorities and explain why they favour them. Other participants can then ask them questions of clarification or contestation to which they can respond. All participants debate the pros and cons of various proposals. The final set of priorities is mutually agreed upon by all participants. Next, brainstorm Actions to Take to run the deliberative workshop(s) below. Deliberative community engagement processes have been used to inform institutional ethics policies on biobanking and benefit sharing. Methods applied in these studies may be a rich resource to draw upon to inform health research priority-setting practice . Also, brainstorm a Strategy to assess whether this level of participation is acceptable to community participants (once specific individuals are invited to participate). Then proceed to Question 6. - If your answer to Question 5 is that community members will be involved as consultants, brainstorm how they will be consulted and how a ratification process involving community members, including those considered disadvantaged or marginalised, can be implemented for the final set of research priorities. Also, consider whether a diverse subset of community partner staff or community members can be selected and trained (as field investigators) to collect and analyse data from community members, and develop strategies for training them. Brainstorm Actions to Take to implement the consultation, ratification, and training processes below. Also, brainstorm a Strategy to assess whether this level of participation is acceptable to community participants. Then proceed to Question 6.	

^a^See: O’Doherty, K.C., Hawkins, A.K., & Burgess, M.M. (2012). Involving Citizens in the Ethics of Biobank Research: Informing Institutional Policy through Structured Public Deliberation. *Social Science & Medicine*. 75, 1604–1611; Marsh, V. et al. (2013). Consulting Communities on Feedback of Genetic Findings in International Health Research: Sharing Sickle Cell Disease and Carrier Information in Coastal Kenya. *BMC Medical Ethics*. 14, 41; Njue, M., Kombe, F., Mwalukore, S., Molyneux, S., & Marsh, V. (2014) What Are Fair Study Benefits in International. Health Research? Consulting Community Members in Kenya. PLoS ONE. 9(12), e113112. doi:10.1371/journal.pone.0113112

### Differences to version 2 of the toolkit

Based on the findings of the 22 interviews with people with lived experience and members of the public who had been engaged in health research and the two case studies, the second version of the toolkit was substantially revised to generate the version presented in this paper. Worksheet 1 was expanded to include questions for selecting not only community partners but also academic partners. The case studies provided rich data on what features community organisations, disabled persons organisations, health service providers, and others can look for to identify good academic partners who can help their communities. That guidance has been incorporated into Worksheet 1.

In light of the case study data, Worksheet 2—Deciding to Partner—was added to the toolkit. The 22 interviews with people with lived experience and members of the public also provided substantial data on the foundations and barriers to their meaningful engagement that informed Worksheet 2 questions and its guidance on how to build missing foundations. Worksheet 3—Deciding to engage with the wider community—(previously Worksheet 2) was also revised substantially in light of interview data on foundations and barriers.

Worksheet 4A (previously Worksheet 3) was revised to incorporate questions related to new sites of power identified from interviews with people with lived experience and members of the public: listening and being heard (Table 2). For example, the worksheet now asks: How will the research team ensure community partners’ and members’ ideas are listened to during consultations and deliberations? Questions at previously identified sites of power were revised in light of new aspects of power-sharing identified by interviews and case studies. For example, interviewees emphasised the importance of having *control* over determining their stage and level of participation. As such, questions related to those two sites of power were revised to state:

- What stage(s) of the priority-setting process will community members be involved in? Is this acceptable to them?
- Will community members be involved as collaborators (decision-makers) and/or consultants? Is this acceptable to them?

The latter question about acceptability was added. New guidance was also added to Worksheet 4A such as about what types of priority-setting mechanisms research teams might use based on the selected level of participation of community partners and members. The different priority-setting mechanism options were drawn from case study data.

Worksheet 4B was added to the toolkit to use in circumstances where community research priorities already exist and priority-setting can thus occur primarily within the research team. Power-sharing should then occur between academic and community partners. What sites of power are relevant in such processes was informed by the W-DARE case study in which priority-setting was largely (but not exclusively) undertaken by the research team.

## Conclusions

Social justice is a foundational moral value guiding health research, policy, and practice. To uphold this core value, global health research projects should ensure the health concerns and knowledge of marginalised communities are reflected in their research topics and questions. Community partners and members should be meaningfully engaged during priority-setting processes (and throughout research projects).

To help assist research teams undertake priority-setting processes where power is more evenly shared with communities, an ethical toolkit (Supplemental Files 1, 2, 3, 4, 5 and 6) has been developed via a three-year program of ethics research. The toolkit is a reflective project planning aid to use before performing priority-setting. Where global health researchers and their partners reflect on and discuss the questions in Worksheets 1 to 4 of the toolkit and then finalise their priority-setting processes’ design, it will create more *inclusive* processes that are less likely to reinforce hierarchies of privilege and subordination. This, in turn, will help deliver projects with research topics and questions that more accurately reflect the healthcare and system needs of communities that are considered disadvantaged or marginalised. Such projects will contribute to the evidence base for reducing global health inequities.

## Supplementary Information

**Additional file 1.** Companion Document

**Additional file 2.** ETHICAL TOOLKIT WORKSHEET 1

**Additional file 3.** ETHICAL TOOLKIT WORKSHEET 2

**Additional file 4.** ETHICAL TOOLKIT WORKSHEET 3

**Additional file 5.** ETHICAL TOOLKIT WORKSHEET 4A

**Additional file 6.** ETHICAL TOOLKIT WORKSHEET 4B

## Data Availability

The datasets used and/or analysed during the current study are available from the corresponding author on reasonable request.

## Abbreviations

CIOMS: Council for International Organizations of Medical Sciences
NIAID: National Institute of Allergy and Infectious Diseases
PLA: Participation for Local Action
W-DARE: Women with Disabilities taking Action on REproductive and sexual health
WOWLEAP: Women with Disabilities LEAP to Social and Economic Progress, Inc.
UNAIDS: Joint United Nations Programme on HIV/AIDS

## Footnotes

1 The first two components of engagement reflect that participation as freedom means having an equal right to shape and define a decision-making space [31]. The latter two components reflect that effective participation is also determined by who is invited to be present and whether they can raise their voice and be heard [7, 21].
