# Supplementary material for: Sharing power in global health research: an ethical toolkit for designing priority-setting processes that meaningfully include communities
Source: Int J Equity Health. 2021 May 25;20:127. doi: 10.1186/s12939-021-01453-y (PMC8145852; doi:10.1186/s12939-021-01453-y)
Supplement: Supplementary file 1 — Additional file 1. Companion Document [file 12939_2021_1453_MOESM1_ESM.pdf]

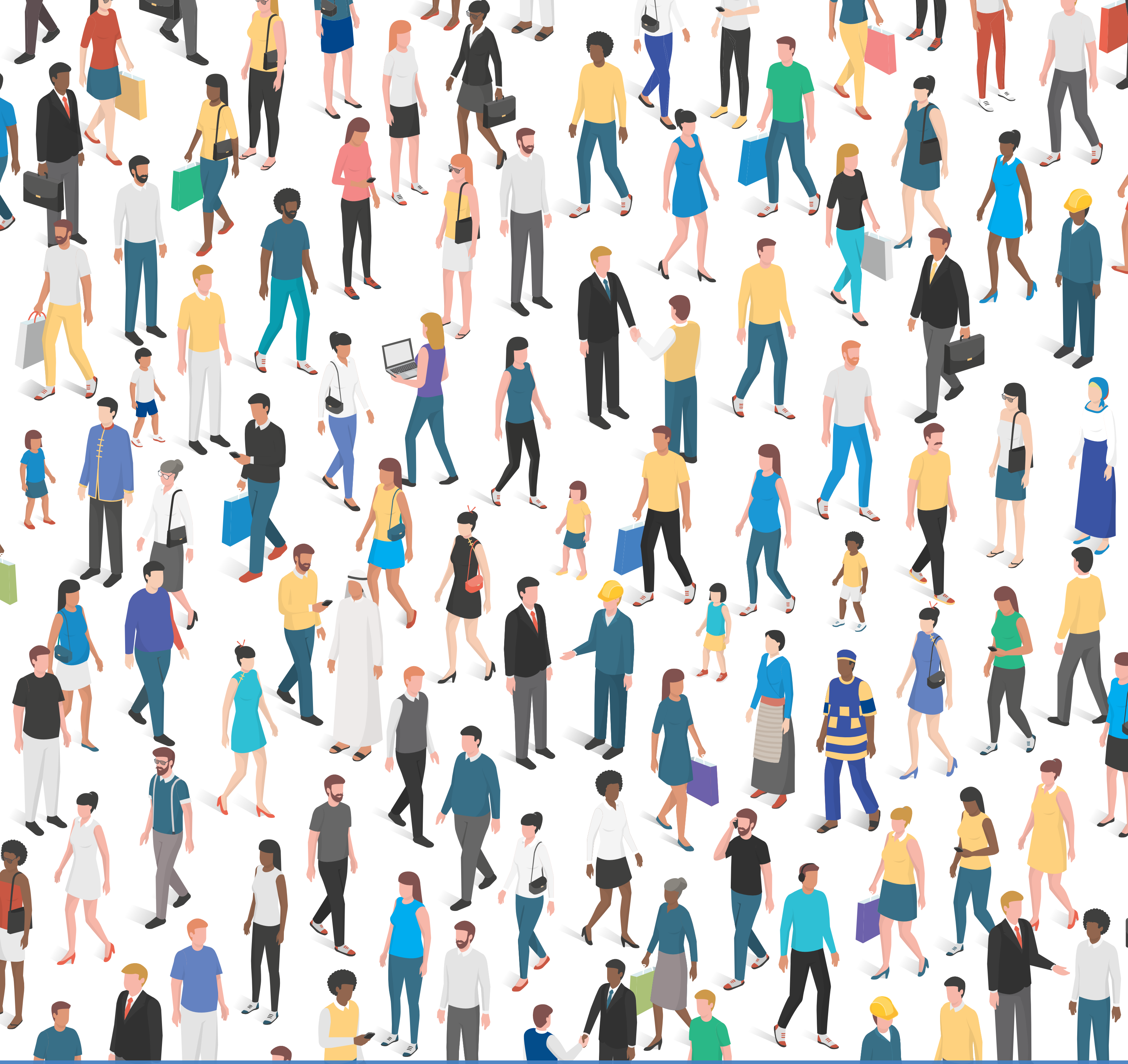

Sharing Power with Communities in Priority-Setting for Health Research Projects: A Toolkit

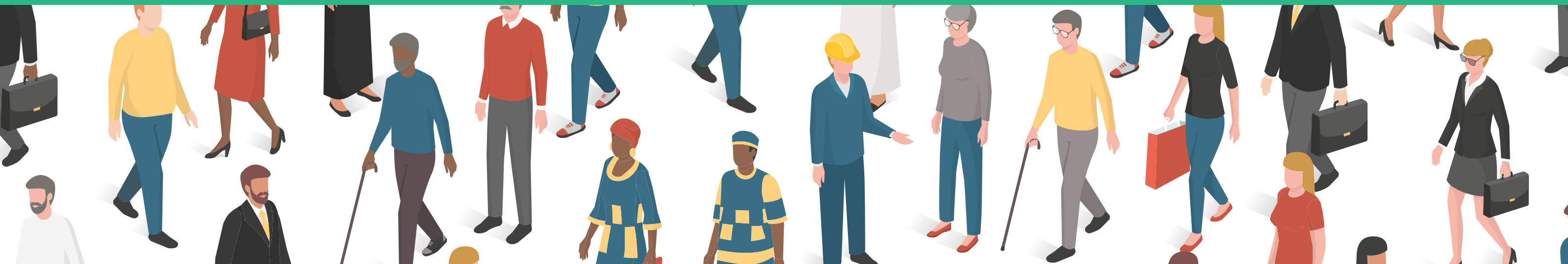

Contents

Preamble 4
Why is the toolkit needed? 4
What is the purpose of the toolkit? 4
What assumptions and values underpin the toolkit? 4
How was the toolkit developed? 5
What is the toolkit? 6
Who should use the toolkit? 6
How should the toolkit be used? 6
Definitions 8
Key Considerations in Worksheet 1: Selecting Partners 9
1. Partners 9
2. Framing 10
Key Considerations in Worksheet 2: Deciding to Partner 11
1. Building Foundations 11
2. Barriers 12
Key Considerations in Worksheet 3: Deciding to Engage with the Wider Community 13
1. Existing priorities 13
2. Building Foundations 13
3. Barriers 14
Key Considerations in Worksheets 4A and 4B: Designing Priority-setting 15
1. Leadership 16
2. Scope 16
3. Empowerment 17
4. Stage of participation 17
5. Level of participation 18
6. Diversity within the community 19
7. Representation 20
8. Mass 21
9. Space 21
10. Ground rules 22
11. Facilitation 22
12. Listening 23
13. Being heard 24
14. Resources and compensation 25
15. Unintended harms 26
16. Accountability 27

Preamble

WHY IS THE TOOLKIT NEEDED?

Helping improve health care and systems for those considered disadvantaged and marginalised is an important goal motivating the conduct of health research worldwide. Their engagement in priority-setting is a key means for setting research topics and questions of relevance and benefit to them.

But communities, especially those considered disadvantaged and marginalised, rarely have a say in the agendas of the very health research projects that aim to help them.

And even where their engagement occurs, without attention to power dynamics, it can lead to tokenism: presence without voice and voice without influence. Voices are excluded from priority-setting, particularly those already disadvantaged or marginalised by their societies' institutions and norms. Existing evidence confirms that, for example, being female, being poor, having little formal education, living with a disability, and/or belonging to certain ethnic groups means community members are listened to less or not at all during health priority-setting.

Where voices from marginalised communities aren't heard, health research projects won't prioritise the key problems they face in accessing and affording health care and services. Without their input, projects are much less likely to generate evidence that will improve health care and systems for them.

It is, therefore, very important to carefully design health research priority-setting processes to share power with communities.

WHAT IS THE PURPOSE OF THE TOOLKIT?

The toolkit aims to help academic researchers and community partners design priority-setting processes that will make the health needs and knowledge of communities, particularly those considered disadvantaged and marginalised, more visible in health research projects' topics and questions. The toolkit is a reflective project planning aid for use before priority-setting is undertaken for a health research project.

WHAT ASSUMPTIONS AND VALUES UNDERPIN THE TOOLKIT?

This toolkit is underpinned by an assumption that the goals of community engagement in health research priority-setting should (amongst other things) promote values of social justice and equity. Two core aspects of social justice are addressing the health needs of those considered disadvantaged and marginalised, and supporting their participation in decision-making. To promote social justice and equity, priority-setting should help make the health concerns and knowledge of such communities visible in health research topics and questions. Priority-setting should also involve their meaningful engagement and participation. This means community members are able to have a say and influence the priority-setting process and its outputs.

A second core assumption of the toolkit is that academic researchers will typically collaborate with community partners (e.g. community organisations, health care providers, policymakers) to conduct projects and engage with the wider community (i.e. community members who are not part of the research team) when setting those projects' research topics and questions. In some cases, academic researchers may initiate a partnership with community partners. In other cases, community partners may initiate a partnership with academic researchers. The toolkit assumes that both academic and community partners will initiate the engagement process with the wider community to set projects' research topics and questions.

A third core assumption of the toolkit is that, while its focus is on priority-setting, community engagement is also important

in other phases of the research process: intervention design, data collection and analysis, and dissemination. Community engagement in priority-setting alone is necessary but not sufficient. Toolkit questions, however, have been designed to inform health research priority-setting. They may apply to community engagement in other phases of research but have not been written with them in mind.

A fourth core assumption of the toolkit is its definition of community. The toolkit takes a broad definition of community. Communities can be based on geography; shared interests or goals; or shared characteristics, situations or experiences, including experiences of marginalisation. They can encompass (amongst others) community leaders and elders, the general public, and people who are part of the health system in that community; namely, patients, health care providers, health care managers, insurers, policymakers, and others.

HOW WAS THE TOOLKIT DEVELOPED?

Development of the toolkit occurred over a three-year period and was led by a bioethicist as part of an Australian Research Council Discovery Early Career Researcher Award. The questions presented in toolkit worksheets were identified based on both conceptual and empirical ethics research. First, six key bodies of literature that discuss participation in contexts of power disparities were analysed for sites of power. Sites of power are features of priority-setting (e.g. ground rules, facilitation) that affect who shapes priority-setting processes, who participates, and who is heard in them. The bodies of literature included development studies, political philosophy, ethics, health priority-setting, public deliberation, and community-based participatory research. Conceptual work was then undertaken to identify ethical considerations related to power-sharing at each site in health research priority-setting. This generated an initial version of the Designing Priority-setting Worksheet.

Sites of power and ethical considerations identified by the conceptual work were then tested against and informed by the experiences and perspectives of researchers, ethicists, community engagement practitioners, community-based organisation staff, and people with lived experience who have been engaged in health research. 51 in-depth interviews and 1 focus group were performed as well as 2 case studies of health research priority-setting processes where communities were involved as partners. Those recruited for in-depth interview came primarily from Australia, the UK, Europe, and Africa, as well as, to a lesser extent, from North America, Latin America, and Southeast Asia. The case studies were of priority-setting processes in India and the Philippines respectively. Based on that empirical work, the Designing Priority-setting Worksheet was revised and the Selecting Partners Worksheet, Deciding to Partner Worksheet, Deciding to Engage with the Wider Community Worksheet, and Companion Document were developed.

In addition to the bioethicist lead (Dr Bridget Pratt), the development of the toolkit has involved several collaborators and research assistants. The two case studies were conducted in collaboration with health systems researchers from the Institute of Public Health (Bangalore, India)—Dr Prashanth N Srinivas and Dr Tanya Seshadri—and public health researchers from De La Salle University (Philippines) and the University of Melbourne—Associate Professors Jesusa M Marco and Cathy Vaughan. Five research assistants from the University of Melbourne, Institute of Public Health, and the University of the Philippines helped to organise and conduct data collection and to assist with data analysis: Natalia Evertsz, Nityasri S N, Jessica Snir, Michelle Walters, Mona Pindog.

WHAT IS THE TOOLKIT?

The toolkit for **Sharing Power with Communities in Priority-Setting for Health Research Projects** is a set of four worksheets and a companion document.

Worksheet 1 helps research teams think about and collectively determine whether they can be strengthened by adding an (or additional) academic or community partner(s).

Worksheet 2 helps research teams reflect on and collectively determine whether they can share power within their partnership.

Once the research team and partnership is finalised, **Worksheet 3** helps its members reflect on and collectively determine whether wider community engagement is necessary in priority-setting and, if so, whether it can be meaningfully done with members of a given community. Where meaningful engagement is necessary and possible, **Worksheet 4A** then helps research teams design the priority-setting process for a given health research project. Reflecting on and collectively answering Worksheet 4A questions will promote the design of priority-setting processes where power is more evenly shared with communities, particularly those considered disadvantaged and marginalised.

Where meaningful engagement is not necessary or possible, **Worksheet 4B** then helps research teams undertake a priority-setting process for a given health research project where power is shared between academic and community partners. The Companion Document provides toolkit users with guidance on how to understand worksheet questions and why they are important.

WHO SHOULD USE THE TOOLKIT?

Academic researchers and their community partners (e.g. community organisations, health care providers, policymakers) should use the toolkit together. This means completing Worksheets 1, 2, 3, and 4 as a team.

Given the toolkit's underlying values and assumptions, it may be especially suited for (but not limited to) use in health research projects that aim to advance health equity and social justice as well as in community-based health research projects.

HOW SHOULD THE TOOLKIT BE USED?

The toolkit is a reflective project planning aid for use before undertaking priority-setting for a health research project. It should be used to develop and inform a final priority-setting plan as follows:

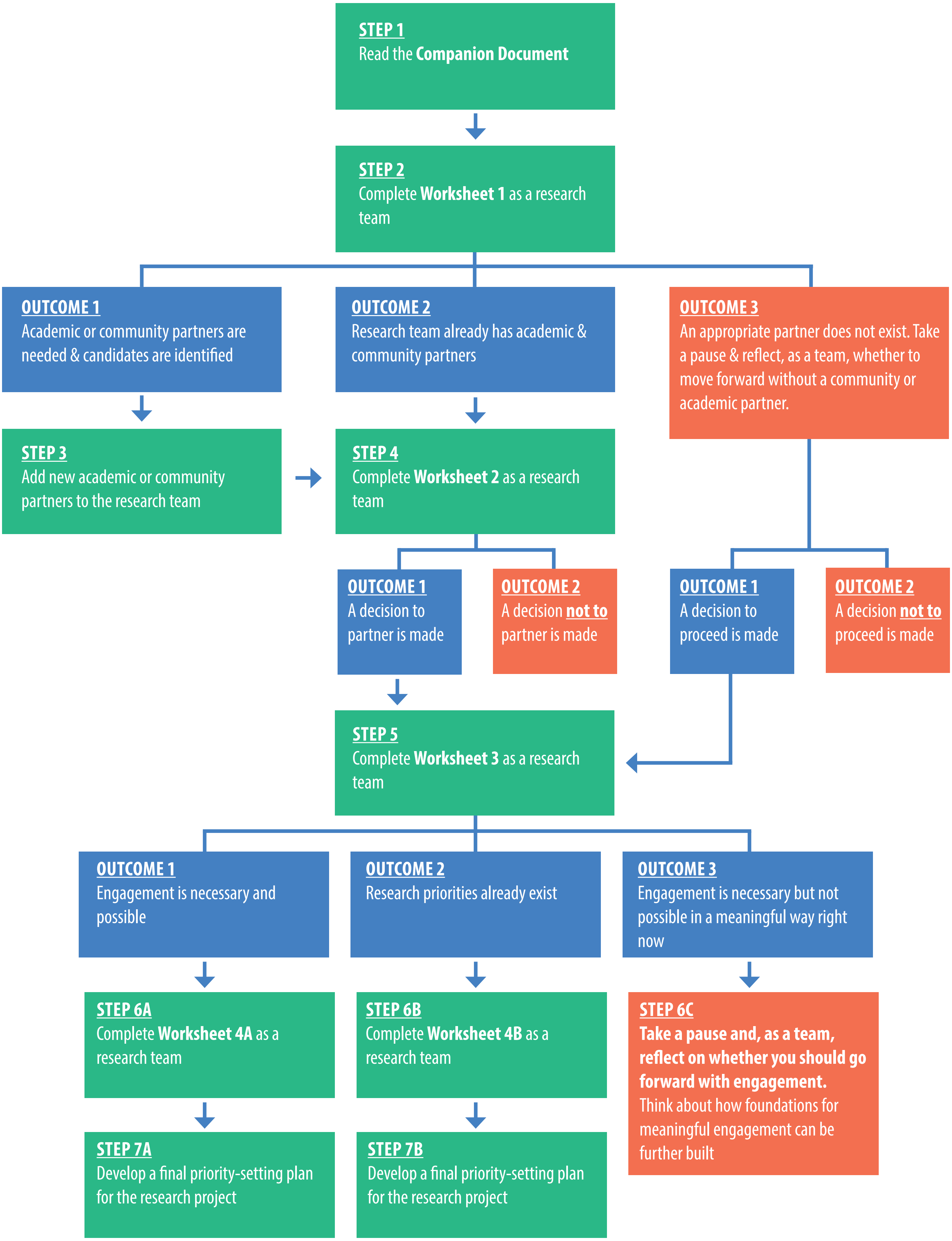
STEP 1
Read the Companion Document
STEP 2
Complete Worksheet 1 as a research team
OUTCOME 1
Academic or community partners are needed & candidates are identified
OUTCOME 2
Research team already has academic & community partners
OUTCOME 3
An appropriate partner does not exist. Take a pause & reflect, as a team, whether to move forward without a community or academic partner.
STEP 3
Add new academic or community partners to the research team
STEP 4
Complete Worksheet 2 as a research team
OUTCOME 1
A decision to partner is made
OUTCOME 2
A decision not to partner is made
OUTCOME 1
A decision to proceed is made
OUTCOME 2
A decision not to proceed is made
STEP 5
Complete Worksheet 3 as a research team
OUTCOME 1
Engagement is necessary and possible
OUTCOME 2
Research priorities already exist
OUTCOME 3
Engagement is necessary but not possible in a meaningful way right now
STEP 6A
Complete Worksheet 4A as a research team
STEP 6B
Complete Worksheet 4B as a research team
STEP 6C
Take a pause and, as a team, reflect on whether you should go forward with engagement.
Think about how foundations for meaningful engagement can be further built
STEP 7A
Develop a final priority-setting plan for the research project
STEP 7B
Develop a final priority-setting plan for the research project

Definitions

Community members: Community membership can be based on geography; on shared interests or goals; or on shared characteristics, situations or experiences, including experiences of marginalisation. Communities encompass (amongst others) community leaders and elders, the general public, and people who are part of the health system in the given community; namely, patients, health care providers, health care managers, insurers, policymakers, and others. Community members are not part of the research team that initiates the priority-setting process.

Community partner: Community organisations, disabled persons organisations, NGOs, persons with lived experience, service users, patients, members of the public, health care providers, and/or policymakers that partner with academic researchers to conduct health research projects. They are part of the research team and are involved as decision-makers in most or all stages of the health research project (unless they choose not to be).

Consultant: Individuals are invited to give their input and to provide information but have no assurance that it will be used by decision-makers to set the health research topic or questions for a given health research project.

Decision-maker: Individuals are part of the group that has the final say on what research topics and questions are selected for a given health research project.

Field investigator: An individual (ideally from the community partner or community) who collects and analyses data from community members as part of consultative priority-setting processes.

Health research priority-setting: Setting the research topic and formulating the research objectives and questions for a health research project.

International researcher: Researchers from universities or other organisations from outside the country where the health research project is being conducted. They are often from high-income country universities or organisations.

Locally-based: Individuals who are in a continuous relationship where they are known to, and trusted by, a given community. They typically share its culture and have lived in or nearby the community for many years.

National researcher: Researchers who are from the country where the health research project is being conducted but are not locally-based in the community where it is being done.

Stepped approach: Facilitation method where small groups with some degree of homogeneity or similar characteristics meet first to deliberate on a topic (e.g. about what research priorities to set). Then those small groups come together as a large group to deliberate about that topic.

Key Considerations in Worksheet 1: Selecting Partners

1. PARTNERS

To consider when you approach people/organisations, or they approach you, to partner in research:

For communities: Can your prospective academic partner(s) help improve the condition of the community you are part of or that your organisation serves?

For academic researchers: Does your prospective community partner(s) represent and can it/they access a community that is considered disadvantaged or marginalised in its diversity?

Community partners could be community organisations, disabled persons organisations, NGOs, persons with lived experience, service users, patients, members of the public, service providers, and/or policymakers.

Why this consideration is important

Working with academic partners can potentially enable community partners to conduct research that is needed by the communities they serve. This often happens when academic partners have certain personal qualities, attitudes, skill sets, and expertise. As an example, being open to listening to people who have different backgrounds and opinions is important. Academic researchers should be:

> **"Open to the idea that communities know their needs much more than we can ever know."**
> [ethics researcher, Kenya]
>
> **"Open minded enough to be responsive in the event that a participatory project opens up new pathways and opens up new possibilities and new ways of doing things. If they aren't then, it's like it's a waste of everybody's time. And borderline, you know and I think offensive."**
> [global health researcher, UK]

Working with community partners can potentially facilitate power-sharing in priority-setting with those considered marginalised and disadvantaged. This often happens where community partners have certain values, capacities, networks, and social standing in their community. As an example, working with a local NGO in comparison to a medical school may potentially make a difference in terms of accessing the voices of those considered to be disadvantaged or marginalised. Medical school staff can often be seen as powerful elites, which may make members of such communities less likely to speak up in front of them. In contrast, NGO staff can often have a reputation for being more community-based and have experience working with such communities.

When working with health care providers or policymakers, it is important to select partners who can access those considered disadvantaged or marginalised within the health system, e.g. community health workers or patients with certain stigmatised illnesses.

2. FRAMING

How will you frame the priority-setting process to the community/academic partner(s) being approached?

Why this consideration is important

Power is more likely to be shared when there is an “open” scope to set health research topics, articulate research questions, and design interventions with partners. Open scope means no topics, or very few topics related to health, are off the table. Ideally, health research partnerships are framed as having an open scope for priority-setting.

But funding and other constraints often make this impossible. In such cases, it is essential to think through, given existing constraints, how to pitch the priority-setting process and partnership in a way that is attractive and of benefit to prospective partners and their communities. It is also important to be transparent about what constraints exist. Transparency can help prevent prospective partners from developing unrealistic expectations about what the priority-setting process and partnership can deliver.

Key Considerations in Worksheet 2: Deciding to Partner

1. BUILDING FOUNDATIONS

How will relationships between partners be built or made stronger?

How will community partners be supported to participate in priority-setting?

Why this consideration is important

Building relationships establishes trust and enhances partners' understanding of one another. It strengthens their ability to see each other's perspectives and their comfort in saying what they think a given project's research topics and questions should be, even when it differs from other partners' views:

> **"Like any relationship that you build with anybody, it's a getting to know you, it's a building trust, it's a sharing more and more in-depth as the relationship develops so that ultimately when you've got that basis of trust there, you can sit across the table and then go actually I really disagree with what you're saying, what I want you to hear from me is this, and you can have far more robust, equal power-sharing relationships."**
> [community engagement practitioner, Australia]

Building relationships further addresses barriers to sharing power in research priority-setting like academic researchers' devaluing of community partners' knowledge:

> **"It also fundamentally shifts the culture within our researchers as they get to know people and respect people to not think well I know everything and you know nothing, and I'm not saying all researchers think like that, but I think it's easy for people that are in an academic, prestigious research position to overestimate the expertise of academia."**
> [community engagement practitioner, Australia]

Academic researchers can build community partners' knowledge of funding processes and research methods and can create priority-setting spaces where community partners feel valued and safe to contribute. Community partners are then more likely to propose research topics and questions, and to share their views and criticisms of others' proposed research topics and questions. Academic researchers can also make reasonable adjustments (e.g. take account of disabilities, write for various literacy levels and language fluencies, have translators) to help ensure that priority-setting activities are accessible and can be performed by all partners.

2. BARRIERS

What barriers still might exist to sharing power between partners?

Why this consideration is important

Barriers can exist at personal, relational, environmental and normative levels that prevent academic and community partners from being involved from the start of priority-setting and/or from being able to raise their voices and be heard during the process. For example, at the personal level, differences in knowledge about research processes and design may make certain partners feel less comfortable speaking up during priority-setting. Academic partners may devalue community knowledge, leading them to invite community partners to participate later in the priority-setting process, rather than from the planning stage. At the relational level, where academic partners play favourites (intentionally or unintentionally), it may exclude certain community partners or make them feel less comfortable sharing their ideas and views during priority-setting. At the environmental level, lack of funding support for engagement pre-grant award may make it difficult to fully involve community partners in priority-setting. At the normative level, where research culture doesn't value different forms of experience and evidence, community partners may be excluded from being involved throughtout and from the start of priority-setting.

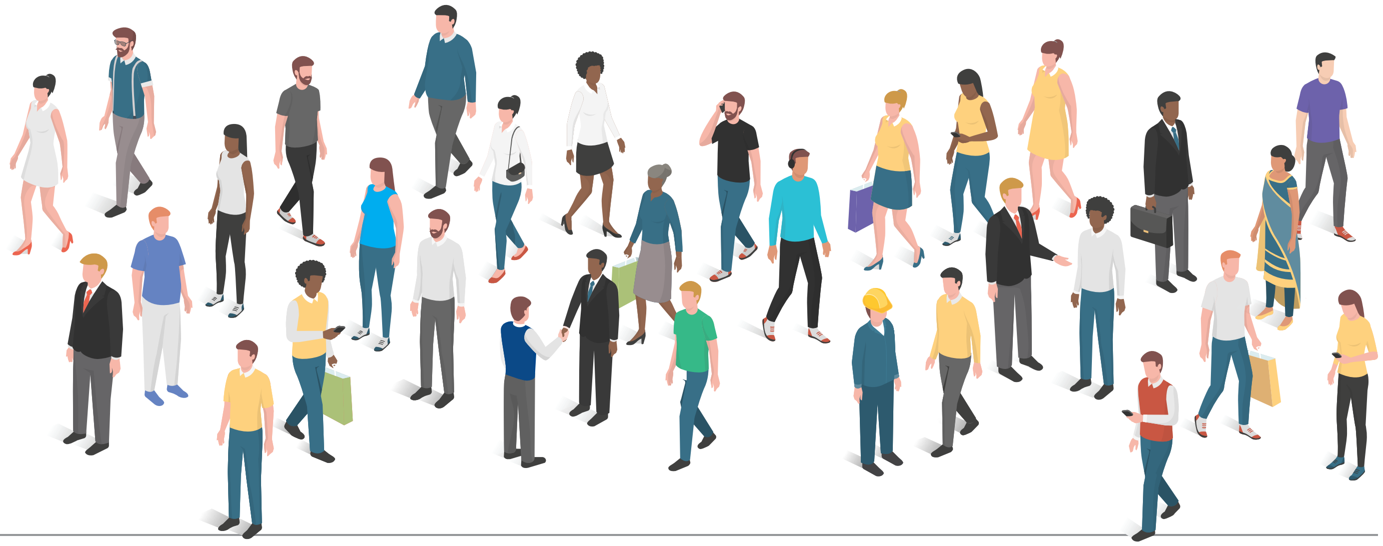

Key Considerations in Worksheet 3: Deciding to Engage with the Wider Community

1. EXISTING PRIORITIES

Have the community's health research priorities, including those of the disadvantaged, less influential, lower status, and/or marginalised, already been voiced?

Why this consideration is important

Where academic partners have been embedded in a given community for a long period of time, it is likely that previous studies have been done where community members' views and ideas, including those of the disadvantaged, less influential, lower status, and/or marginalised, have been sought and speak to or explicitly articulate their health research priorities. Where community health research priorities have already been articulated and documented, then it may not be necessary to undertake community engagement activities in priority-setting for new projects. Doing so, in fact, might comprise a poor use of resources that could be better spent on other research activities or engagement in other phases of the research process (e.g. data collection, data analysis, dissemination).

2. BUILDING FOUNDATIONS

How will relationships between the research team and the wider community be built or made stronger before priority-setting starts?

How will community members be supported to participate in priority-setting?

Why this consideration is important

Strong relationships may already exist between community partners and their communities. Even so, building or strengthening relationships between them and between academic partners and communities can help a wider range of community members become aware of engagement opportunities in health research. This, in turn, can promote greater diversity amongst those engaged during priority-setting. Building relationships can also empower those who are engaged in health research to feel comfortable sharing their stories and vulnerabilities, and to be critical during priority-setting . **Although people's vulnerabilities are the hardest to draw out, these are key stories for agenda setting. Research teams can identify those problems that pain people the most and pursue them in research.**

Research teams can build community members' knowledge of research and priority-setting and create spaces where they feel comfortable and valued. The latter is essential to capture the voices of those who have had:

> "intense personal experiences of all their power stripped away…I think there is a feeling of powerlessness that those experiences leaves that you bring with you when you come and sit at the table. And so if we genuinely wanna hear those voices I think we have to go the extra mile to make it a safe space and encouraging space for them to feel that their voices have value and they can be heard."
> [community engagement practitioner, Australia]

Creating a safe space helps make those engaged feel less intimidated by academic partners and reduces feelings of internalised powerlessness (i.e. feeling that they know little relative to 'expert' academic researchers). It can also make them feel more comfortable sharing their stories and vulnerabilities.

3. BARRIERS

What barriers to meaningful engagement with the wider community might still exist?

Why this consideration is important

Barriers can exist at personal, relational, environmental and normative levels that prevent community members from being present for priority-setting and/or being able to raise their voices and be heard during the process. For example, at the personal level, academic researchers or policymakers may not have much experience with engagement and/or devalue community members' knowledge:

> **"Their view of co-design was far more tokenistic than mine; you kind of read the rhetoric around involvement and they go oh yeah, yeah, yeah, we want that but they don't really know how to do it, and they also don't really appreciate that they have to give up power to do it."**
> [community engagement practitioner, UK]
>
> **"I was the only patient advocate in a room of thirteen people, and there was a person there representing a fairly high level of healthcare who obviously didn't feel the need for a patient in the room. And, I felt that animosity, you know."**
> [person with lived experience who has been engaged in health research, UK]

At the relational level, where communities have previously been exploited by academic researchers, this comprises a substantial barrier to meaningful engagement. The resultant lack of trust makes it especially difficult to engage community members, including those considered disadvantaged and marginalised, to participate in priority-setting. Where community partners' networks don't reach those considered marginalised within the wider community, they can be excluded, thereby reducing the diversity of those engaged from a given community.

Broader environmental factors also affect whether power sharing is possible in health research priority-setting. For example, if engagement policies do not require compensation be provided at adequate levels to those engaged, it is very difficult for community members to participate in priority-setting processes. At the normative level, in countries where there is a strong tradition of civil society, engaging communities in research priority-setting is more likely to be feasible. In countries where there is limited civil society, or where decision-making power is delegated to authorities who are then seen to speak for community members, it is much more challenging to achieve such engagement.

Key Considerations in Worksheets 4A and 4B: Designing Priority-setting

Priority-setting processes consist of 4 main components: who initiates, for what purpose, who participates, and how they participate during the process. The considerations in Worksheets 4A and 4B relate to these different components of priority-setting, as depicted below (Figure 1). Two considerations are overarching and do not pertain to specific components of priority-setting: resources and compensation, and unintended harms.

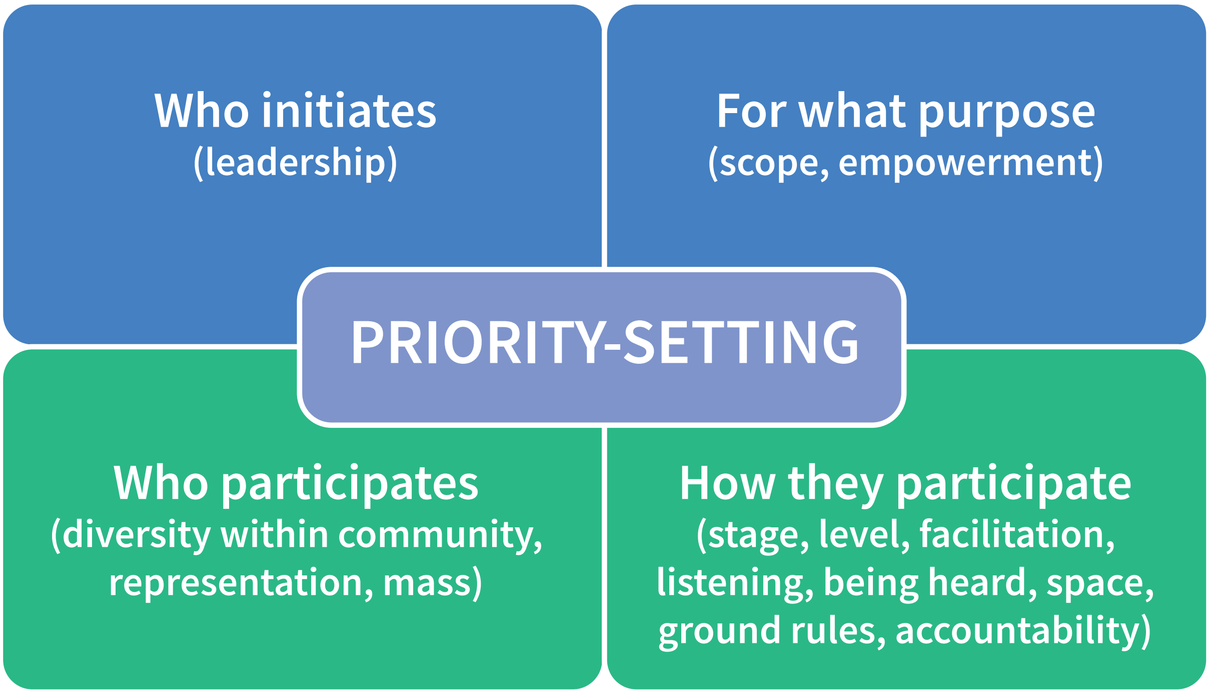

Figure 1: Components of priority-setting and their associated ethical considerations

If completing Worksheet 4A, please read considerations in green and blue. If completing, Worksheet 4B, please read considerations in green and orange.

Considerations in blue apply to priority-setting between research partners. Considerations in orange apply to priority-setting with the wider community. Considerations in green apply to both types of processes.

Key Considerations in Worksheets 4A and 4B: Designing Priority-setting

1. LEADERSHIP

Who will lead the health research priority-setting process?

Why this consideration is important

A distinction is made in the development studies literature between ‘invited spaces’ and ‘created spaces’. Invited spaces are those into which people are invited to participate by various kinds of power-holders or authorities. Created spaces are those that are claimed by the less powerful from power-holders or created by the less powerful. They are often spaces that emerge organically out of common concerns or identifications experienced by those marginalised by social institutions and norms.

The implication of these concepts for engagement in health research priority-setting is, first, to recognise that, in practice, such engagement is typically an invited space, where international and national researchers invite locally-based researchers, community partners, and (more rarely) community members to participate. Two types of power dynamics are then often recreated within such spaces—namely, those of:

- ‘expert’ academic researchers over community partners and members; and
- international or national academic researchers, who “parachute” in from a foreign university or the capital city, over locally-based academic researchers, community partners, and community members.

To avoid reinforcing such power dynamics, it is important that locally-based academic researchers and community partners are amongst those leading health research priority-setting, including any engagement done with the wider community as part of it.

2. SCOPE

Will research topics be solicited relating to *all* health problems experienced by community members?

Why this consideration is important

Power is more likely to be shared when there is an “open” scope to set health research topics, articulate research questions, and design interventions with partners. Open scope means no topics, or very few topics related to health, are off the table. Ideally, health research partnerships are framed as having an open scope for priority-setting.

But funding and other constraints often make this impossible. In such cases, it is essential to think through, given existing constraints, what health problems can be the focus of priority-setting, e.g. a broad range, several specific ones, one specific one, etc. When explaining the priority-setting process to community members, it is crucial to be transparent about what health problems can and cannot be the focus of priority-setting and subsequent research projects, and why. Transparency can help prevent community members from developing unrealistic expectations about what the priority-setting process can deliver.

3. EMPOWERMENT

Will community partners be empowered as researchers during priority-setting?

Will community members' capacity to participate in research priority-setting be strengthened?

Why this consideration is important

Two main types of goals exist for community engagement: instrumental and transformative. In health research, community engagement is often discussed as having an instrumental goal—namely, as being a means to enhance the relevance of research questions to communities and to promote research translation by generating buy-in from policymakers, healthcare providers, and other key stakeholders.

Less common are engagement processes that aim to empower community partners and members. Empowering community partners as researchers means building their knowledge, confidence, networks, and capacities to undertake research, including priority-setting, on their own. Empowering community members to be involved in research priority-setting means enhancing their knowledge, confidence, networks, and capacities to participate in such processes. These affective and cognitive changes can be built up over research cycles (e.g. one priority-setting process to the next) and can ultimately lead to behaviour changes that challenge unequal power relationships. For example, changes where a community partner identifies its own research priorities and then requires all studies done with it to align with those priorities.

Where engagement in research priority-setting only has instrumental purposes, this limits its capacity to reduce perceived inequalities in power between academic researchers and communities. It will fail to empower community partners and their communities in ways that support their right to help set research agendas and is, therefore, not ideal.

4. STAGE OF PARTICIPATION

What stage(s) of the priority-setting process do community partners want to be involved in?

What stage(s) of the priority-setting process will community members be involved in? Is this acceptable to them?*
*To ascertain once specific community members are invited to participate.

Why this consideration is important

The stage in decision-making at which individuals begin to participate is significant. Earlier entry is associated with higher quality participation. It means making a greater number of decisions, including those that determine how the priority-setting process is organised. Being part of more stages in the priority-setting process is also associated with higher quality participation. Individuals have a greater opportunity to raise their voice and be heard.

In priority-setting for health research projects, the *stages of participation* are:

1. Conceptualising the priority-setting process,
2. Planning the priority-setting process,
3. Research topic solicitation,
4. Research topic prioritisation, and
5. Formulating the research objectives and question(s) and interventions.

Key Considerations in Worksheets 4A and 4B: Designing Priority-setting

The start of the priority-setting process is *conceptualising* it. Conceptualising the process encompasses setting the aims and methods to generate research topics and questions. Planning how the priority-setting process will be performed entails designing any data collection tools and determining how to implement the priority-setting methods. Research topic solicitation means coming up with a list of possible health problems or needs upon which to focus the research project. In some instances, this may entail collecting and analysing data to generate a list of health problems or needs. Research topic prioritisation means selecting a single health problem or need from that list to be the project's focus. Research objectives, question(s), and/or actions to take (interventions) for the project are then developed that relate to the selected health problem or need. These objectives and questions reflect what knowledge needs to be collected about the health problem to better understand and/or determine how to address it. Interventions are strategies or activities to address the health problem or need.

Reducing perceived power inequalities would support the early entry of locally-based researchers, community partners, and community members, including those that are considered disadvantaged, less influential, lower status, and/or marginalised. It would also support their being involved during all, or at least several phases, of the priority-setting process.

At the same time, it is also essential that community partners and members be able to make decisions about the nature of their engagement. Sharing power means they should have control over what stages of the priority-setting process they are engaged in.

5. LEVEL OF PARTICIPATION

Do community partners want to be involved as collaborators (decision-makers) or consultants?

Will community members be involved as collaborators (decision-makers) and/or consultants? Is this acceptable to them?*

*To ascertain once specific community members are invited to participate.

Why this consideration is important

Participants' entry point into a decision-making process does not exclusively determine the quality of their participation. Their *level* of participation also matters. A variety of levels of participation exist, with some more active and influential than others. Sherry Arnstein (1969) and others distinguish between the following levels of participation: collaboration, consultation, and information-giving.

Collaboration involves shared decision-making between academic partners and community partners or between the research team and community members. Both parties deliberate together and their knowledge is integrated during priority-setting. The final research topic and questions are an explicit product of deliberation that is endorsed by all participants. They are a ratified collective conclusion and reflect the voices of the research team and community members.

Consultation is characterised by community partners or members being invited to give their input (often through interviews or focus groups) but having no assurance that it will be used. Where community partners are consulted, academic partners have the power to interpret the content of their interviews or focus groups. Academic partners construct possible research topics and questions using that content and decide which to study. Where community members are consulted, the research team has the power to interpret the content of their interviews or focus groups. Here, the final research topic and questions are an inferred product constructed and selected by the research team.

Informing means community partners or members are told the outputs of priority-setting for research projects but do not have a say in the process or decisions in any way.

Power is more evenly shared where community partners and members participate in health research priority-setting as collaborators (decision-makers). Where they are collaborators, priority-setting processes are more likely to bring different ways of knowing and perspectives together to create outputs that reflect a diversity of people's knowledge. However, giving community partners and members control means they should decide their level of participation in priority-setting.

6. DIVERSITY WITHIN THE COMMUNITY

6a. Which community roles will you engage during priority-setting and for what reasons?

6b. List which of the roles identified in Q6a correspond to greater or lesser influence and status within the community.

6c. Who are considered disadvantaged, less influential, lower status, or marginalised within these roles?

6d. Which of those groups or stakeholders in Q6c will you engage and for what reasons?

6e. Is it fair to bring these community members into the same decision-making space?

Why this consideration is important

Ideally, sharing power demands a *range* of community members are present or represented during health research priority-setting so that processes are informed by differently situated actors.

Achieving range means that participants span a wide spectrum of roles in a community and include those considered disadvantaged, less influential, lower status, or marginalised within the community. Pooling the knowledge of all social positions maximises the information base used to set health research priorities. Listening to others who speak from different, less privileged perspectives will also lead participants to transform their opinions on what research priorities should be. It will transform them from narrow expressions of self-interest to a more complete account of what research is needed to serve the population as a whole, especially those considered disadvantaged and marginalised.

Community roles that might be relevant to include in health research priority-setting are:

- community leaders,
- patients,
- people with lived experience of illness or disability,
- families and carers,
- the general public,
- health care providers and
- policymakers.

Each role likely brings knowledge or experience that will inform the health research agenda. Their inclusion may also promote the priority-setting process having impact (because participants have the power to take the research agenda forward) and/or legitimacy (because participants' presence gives the outputs of health research priority-setting credibility).

Roles of lower influence or status may often be the general public and patients. In terms of who might be considered disadvantaged, less influential, lower status, or marginalised within these roles, some examples are given below:

- Community leaders considered less influential or less likely to have a voice might be women or those living in remote areas.
- Policymakers considered less influential might be district or village-level policymakers (compared to Ministry of Health staff).
- Community members considered to be disadvantaged might be individuals living in settlements with less land or animals, or who grow crops generating less income.
- Community members considered to be marginalised might be groups that experience stigmatisation and/or oppression.
- Health care providers and patients considered marginalised might be community health workers (compared to doctors and nurses) and patients with certain stigmatised illnesses.

Although the research team may have decided community members should be engaged as collaborators, this may need to be reconsidered or revisited in some situations. In certain contexts, engaging community members as consultants may be necessary because community members cannot be brought together in the same deliberative fora for ethical reasons or due to cultural norms. For example, local administrators can be powerful oppressors of the LGBTQ community and their access to health services. It would arguably not be desirable for them to participate in the same deliberative forum as members of the LGBTQ community.

7. REPRESENTATION

Which organisations or individuals will represent the roles listed in Q6a?

Do these representatives encompass those considered disadvantaged, less influential, lower status, or marginalised within each role, as identified in Question 6d?

Why this consideration is important

Once roles to engage in priority-setting are identified, determining who should represent them is essential. Two types of representatives are: 1) organisations and 2) individuals.

Selected representatives should genuinely reflect the needs and interests of the role they are representing in a way that is accountable. They should encompass those considered disadvantaged, less influential, lower status, and/or marginalised within each role to ensure that participants in priority-setting reflect the community's diversity.

Desirable features of organisational representatives include their having a systemic practice of consulting members on their needs and priorities, having a systemic practice of reporting back to members, and having members who reflect the diversity of the role or group they represent, including those considered disadvantaged, less influential, lower status, and/or marginalised. For patient organisations, this could mean having members that span different genders, levels of education, ethnicities or castes, levels of income, residences (urban and rural), and employment statuses (formal and informal).

Desirable features of individual representatives include living a similar reality to those they are representing; collectively reflecting the diversity of the role or group they represent, including those considered disadvantaged, less influential, lower status, and/or marginalised; being able to think from the perspective of others in their community or with their role, particularly those who are considered disadvantaged or marginalised; and being able and willing to relay information back to those they represent.

8. MASS

Will the number of community partner staff and academic partner staff participating in priority-setting be similar? Will similar numbers of different community partners participate? If not, what are the reasons?

Will the number of community partner staff and community members be greater or equal to academic partner staff in consultations and deliberations during priority-setting?

Will the number of representatives of lower status community roles (identified in Q6b) be sufficient at consultations and deliberations during priority-setting?

Why this consideration is important

To ensure that the powerful and/or those who typically have a voice in health research priority-setting do not dominate priority-setting by force of numbers, it is necessary to balance power through equality or slight inequalities in numbers. When priority-setting occurs within the research team, it is essential that similar numbers of academic and community partner staff participate. Where the research team has several community partners representing different segments of a community, they should also be equally represented. For example, where different community partners represent individuals living with different types of disabilities, having similar numbers of their staff present will mean that priority-setting involves similar numbers of individuals living with mobility, visual, hearing, cognitive and psychosocial impairments.

When priority-setting is with the wider community, it is essential that equal or more numbers from the community participate than academic researchers. Although lower status roles will vary by setting, balancing power might, for example, entail having equal or more patient and public representatives to policymaker and community leader representatives. Achieving sufficient numbers of stakeholders who are considered disadvantaged, less influential, lower status, and/or marginalised is also important. This will also vary by setting. For instance, in communities where women are considered less influential or lower status than men, it could mean having equal or greater numbers of women relative to men present at consultations and deliberations during the priority-setting process.

9. SPACE

Where will you hold the priority-setting process for your research project?

Why this consideration is important

The physical spaces selected for health research priority-setting can promote or obstruct the presence and voice of community partners and members, particularly those considered disadvantaged, less influential, lower status, and/or marginalised. Where spaces are physically difficult for community partners or members to access, it can result in their not being present for priority-setting. Where spaces are imbued with certain norms, behaviours, and languages, people favoured by those norms or more practised in those behaviours and languages will dominate priority-setting. When priority-setting processes are held at research institutions or the Ministry of Health, for example, community partners and members may be less likely to raise their voices.

Holding priority-setting in spaces that community partners are familiar with and comfortable in can promote their voices being raised; holding priority-setting in spaces within the community can promote community members' voices being raised. Nevertheless, local spaces may also be characterised by exclusionary norms, for example, where minorities are simply informed and generally do not have a say. Priority-setting using such spaces will likely mean those considered disadvantaged or marginalised within the community are present but rarely speak.

10. GROUND RULES

Will community partners and members be involved in developing and approving the ground rules for the priority-setting process? If not, what are your reasons?

What ground rules will be included to ensure community partners have an equal opportunity to speak relative to academic partners, are listened to, and are heard during priority-setting?

What ground rules will you include to ensure stakeholders identified in Question 6d aren't silenced during priority-setting?

Why this consideration is important

What ground rules are set for the priority-setting process have a significant impact on who is present and whether their voices can be raised and heard. Ground rules specify who can and can't be participants, who can speak, what languages are used, how different participants' views are used, and how a decision or closure is reached in priority-setting.

Power is more likely to be shared where ground rules are developed with community partners and members and where they promote the inclusion of those considered disadvantaged, less influential, lower status, or marginalised in priority-setting. For example, priority-setting might include ground rules that give community partners and members more time to speak, privilege ways of speaking like storytelling and rhetoric, affirm that everyone has an equal right to speak, require people to take turns speaking, disallow interrupting, disallow failing to return to parked issues, and clearly define how community partners' and members' views and ideas will be used to set research topics and questions.

Transparency is important to build community members' trust in the research team and the legitimacy of the health research priority-setting process. Ensuring ground rules are disclosed and fully explained to community members who participate in priority-setting also promotes their understanding of how the process will work and thus their capacity to raise their voices.

11. FACILITATION

Will you have a locally-based person facilitate consultations and deliberations during priority-setting? If not, what are your reasons?

How will the facilitation method/approach give participants an equal opportunity to speak at focus groups and deliberations during priority-setting?

How will the facilitation method/approach make community partners and members feel comfortable sharing relevant, personal stories about their community's health concerns during priority-setting?

Why this consideration is important

Good facilitation is critical because priority-setting spaces are not neutral. Asymmetrical power dynamics exist within them: between research partners, between research teams and community members, and amongst community members. Such power dynamics can result in certain individuals being excluded from or feeling uncomfortable raising their voice and being heard.

A certain approach to facilitation is necessary to make sure all voices get the same opportunity to be heard during interviews, focus groups, and deliberations undertaken as part of priority-setting. Very careful set up is necessary in most contexts to make community partners and members, especially those considered disadvantaged, less influential, lower status, and/or marginalised, comfortable sharing and to draw out their voices.

Sharing vulnerabilities was identified as especially key for health research agenda setting:

> **"people don't want to share their pain, they don't want to share their vulnerability, they wanna conceal it. But pain often brings about purpose. So the thing that pains you is the antithesis that you need to pursue."**
> [person with lived experience who had been involved in research, Australia]

Although people's vulnerabilities are the hardest to draw out, those things that pain people the most are what need to be pursued in research projects. Community partners and members must feel comfortable sharing their vulnerabilities so the problems that pain people in their community the most can be identified and pursued in research.

12. LISTENING

How will the research team ensure community partners' and members' ideas are listened to during the priority-setting process?

Why this consideration is important

Listening to what community partners and members, including those considered disadvantaged, less influential, lower status, and/or marginalised, say in health research priority-setting helps ensure that their voices are captured and considered. It further demonstrates that their views are respected and valued. There are several key aspects to listening:

- Dialogue: Asking questions and for clarifications, especially on points where you disagree; Feeding back (i.e. this is what I think you said…); Considering and responding to people's comments
- Taking turns: Speaking one at a time; Avoiding talking over people or interrupting them
- Documentation: All comments and views are written down or recorded during priority-setting consultations and deliberations, including who said them

Like facilitation, documentation of priority-setting must be done in a particular way to share power with community partners and members. It should be performed by community partners or members, wherever possible, rather than academic partners. Its outputs should be made available to participants in local languages and a mechanism for participants to review those outputs should exist.

13. BEING HEARD

Will community partners' voices have equal or greater weight in the priority-setting process relative to academic partners' voices?

Will the voices of community members, especially those considered disadvantaged, less influential, lower status, and/or marginalised, have equal or greater weight than other participants' voices when setting research priorities? If not, what are your reasons?

Why this consideration is important

Where research priorities are products of deliberations, the inputs of academic partners, community partners, and community members should, at a minimum, be treated equally. The views of community partners and members are taken on board. A joint product is created with inputs from academic partners and community partners or with inputs from the research team and community members:

> **"your views have been acknowledged and woven into the equation. And you've had some influence in determining some of the priorities, or how the priorities were voiced."**
> [person with lived experience who has been involved in research, UK]

However, where voices have historically been excluded, privileging them is often the just course of action. This would demand giving locally-based academic researchers', community partners', and community members' views and perspectives greater weight in priority-setting. They should have more of a say in what research topics and questions get set for a given research project than external academic researchers.

Where research priorities are set after consultations, academic partners or the research team will typically control the translation of knowledge gained from consultations with community partners or members into research priorities. When doing so, it is essential that they use the views expressed by community partners and members, especially those considered disadvantaged, less influential, lower status, and/or marginalised, to construct the given project's research topic and questions. Otherwise, community members' voices won't be heard. They should also:

1. Be transparent about what information they have and haven't used from community partners and members.
2. Provide a justification for what they have used versus what they haven't used.

14. RESOURCES AND COMPENSATION

How will community partners and communities be compensated for participating in priority-setting?

Will community partners have control over any project resources?

Will full information about the research project's budget be disclosed to community partners?

Why this consideration is important

It is essential to compensate community partners and communities for participating in priority-setting. Priority-setting processes can be time-consuming and involve significant amounts of work. It is unfair and disrespectful to expect community partners, key informants, field investigators, facilitators, and community members to contribute without compensation. It is also less likely that they will be able to participate because the lost work hours and income will be too large for it to be feasible.

For community partners, community organisation staff members' time should be compensated at their pay rates within their organisation and individuals unaffiliated with an organisation should be employed by an academic partner (in their country), put on a contract, and paid at an appropriate rate. Fixed term contracts are preferable to casual contracts, which are less secure and typically do not come with benefits (e.g. sick leave, superannuation).

For field investigators and facilitators, best practice also means compensating them as per a normal job, i.e. putting them on contract and paying them at an appropriate rate.

For key informants and community members, compensation should cover but not exceed their time and transport. In some instances, where community members participate during work hours for which they receive a salary, they may not need or want compensation and it is important to ask them whether they do. It may not be necessary to provide compensation in such cases.

Additionally, sharing power in priority-setting means sharing control over project resources and being transparent about them with community partners. Community partners should be involved in setting the research project budget. Where community partners are leading activities or stages of priority-setting, they should be given project resources to spend to carry them out. To achieve transparency, community partners should be provided with a copy of the research project's budget (as awarded or as in the grant submission if pre-award) and given an explanation of what the different line items in it mean.

15. UNINTENDED HARMS

What harms do you think might result from the priority-setting process?

Why this consideration is important

Health research priority-setting processes that engage community members have the potential to generate harms for their well-being. It is, therefore, important to try and anticipate what harms could result from the priority-setting process. If significant harms are identified, revisions to the priority-setting process design should be made. Examples of possible harms that could eventuate from a priority-setting process are:

- Disruption of community power dynamics leads to negative repercussions for members of already stigmatised and marginalised groups.
- The safety of certain groups with illegal status is compromised.
- Some community members experience disrespect from other community members during priority-setting.
- Community members are labelled as marginalised or having lower status roles.
- Community members are taken away from work or other responsibilities for too much time.
- The community partner's relationships with community leaders and/or members are negatively affected.
- Community members develop unrealistic expectations about what the research can deliver.

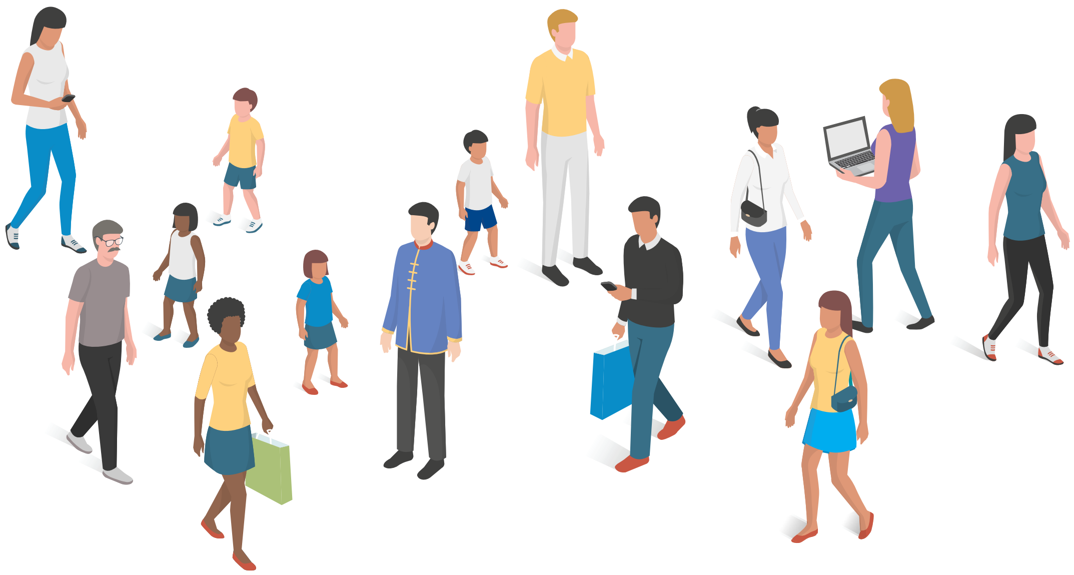

16. ACCOUNTABILITY

How are academic and community partners going to evaluate research partners' participation in the priority-setting process?

Has the wider community endorsed the selected research topic and questions?

Will the research team act upon the final research topic and questions?

How will the final research topic and questions be fed back to field investigators and community members, including those considered disadvantaged, less influential, lower status, and/or marginalised, after priority-setting?

How are the research team and community members going to evaluate community members' engagement in the priority-setting process?

Why this consideration is important

Accountability is important for ensuring community partners' and members' voices are heard and building the legitimacy of the priority-setting process. It means that participants and their community have the right to hold the research team to a set of standards, to judge whether the research team has fulfilled its responsibilities, and to take measures if they determine that these responsibilities have not been met. It also means that research team members have the right to hold each other accountable to certain standards and responsibilities.

Three responsibilities of the research team are: 1) feeding back, 2) acting on research priorities, and 3) evaluation. Feeding back means sharing resultant research priorities with consulted community members and giving them an opportunity to comment on the priorities. In cases where the research team did not undertake a new priority-setting exercise, it is useful to get community approval or feedback on the topic selected by the research team before starting the new research project.

Achieving action on the research priorities ensures that community partners' and members' voices are heard. It means that research projects focusing on the identified priorities are funded and performed. Finally, accountability as evaluation means developing benchmarks for power-sharing in the priority-setting process and assessing whether they were met after priority-setting is completed. Benchmarks could include achieving a diversity of participants and getting community partners' and members' voices heard. Where evaluations demonstrate that community engagement in priority-setting fell far short of power-sharing, it then raises questions about whether it is ethical to take resultant research priorities forward.
