# Supplementary material for: Sharing power in global health research: an ethical toolkit for designing priority-setting processes that meaningfully include communities
Source: Int J Equity Health. 2021 May 25;20:127. doi: 10.1186/s12939-021-01453-y (PMC8145852; doi:10.1186/s12939-021-01453-y)
Supplement: Supplementary file 2 — Additional file 2. ETHICAL TOOLKIT WORKSHEET 1 [file 12939_2021_1453_MOESM2_ESM.pdf]

ETHICAL TOOLKIT WORKSHEET 1

Selecting Partners Worksheet: Questions for Reflection and Discussion

This worksheet should be completed by the research team. Please first read the **Companion Document: Key Considerations in Worksheet 1** and then complete Worksheet 1. To complete Worksheet 1, reflect on and discuss Questions 1 and 2 as a team. For each question, record your team's answer, read the Next Steps to take, identify Strategies and/or Actions to Take, and record them below.

1. PARTNERS

For communities: Can your prospective academic partner(s) help improve the condition of the community you are part of or that your organisation serves?

For academic researchers: Does your prospective community partner(s) represent and can it/they access a community that is considered disadvantaged or marginalised in its diversity?

Community partners could be community organisations, disabled persons organisations, NGOs, persons with lived experience, service users, patients, members of the public, service providers, and/or policymakers.

TEAM ANSWER

NEXT STEPS

- If your answer is **yes**, move to Question 2.
- If your answer is **no**, **Brainstorm** possible candidate academic researchers with whom to partner. See below for suggested personal qualities, attitudes, skill sets, and expertise to look for in academic partners. Alternatively, **Brainstorm** possible candidate individuals or organisations from the community with whom to partner. See below for suggested qualities, attitudes, networks, capacities, and values to look for in community partners. Then move to Question 2.
- If **no appropriate partners** can be identified, take a pause and discuss, as a research team, whether it is appropriate to move forward without a community or academic partner. If the answer is yes, move to Worksheet 3.

BRAINSTORM CANDIDATE PARTNERS

Features to guide the selection of academic and community partners:

FEATURES OF A GOOD ACADEMIC PARTNER:

- ☐ Passionate about co-design, especially any senior researchers who are approached
- ☐ Able to include community engagement practitioners in the research team or have access to them at their university or institution
- ☐ Sensitive to community problems
- ☐ Embedded in the community
- ☐ Service minded: aim to benefit the community
- ☐ Willing to share decision-making and to go with community partners' suggestions
- ☐ Willing for community partners to lead priority-setting and/or the research project with them or on its own
- ☐ Share guiding values with community partners (e.g. equity, social justice)
- ☐ Open to sharing personal information to develop deep relationships with community partners
- ☐ Open to listening and considering different opinions, to learning about each other
- ☐ Recognise and value community partners' capacities and expertise
- ☐ Trained in patient and public involvement, co-design, community engagement, etc.
- ☐ Good communication skills
- ☐ Good conflict resolution skills

FEATURES OF A GOOD COMMUNITY ORGANISATION PARTNER:

- ☐ Have leaders, staff, and members from the community that reflect its diversity, including those considered disadvantaged or marginalised (e.g. women, youth, LGBTQ, poor, remote, living with different types of disability)
- ☐ Membership coverage reaches entire (geographic) scope of community
- ☐ Decision-making processes are participatory and rely on deliberative norms (e.g. equal opportunity to speak, question and answer, consensus, dissent expressed and discussed)
- ☐ Have strong networks with the community, including those considered disadvantaged or marginalised within it, through grassroots work and outreach
- ☐ Treat members equally, prioritise their needs equally, address their concerns and interests equally
- ☐ Have political savvy and networks (i.e. expertise in the politics of the context/country and relationships with policymakers)
- ☐ Share guiding values with academic partner
- ☐ Understand research and may have some research training (e.g. staff with research degrees)
- ☐ Have capacity (time, staff) to be partners
- ☐ Likely to buy into the project (e.g. its agenda aligns with set topic of project, it is open to the topic)
- ☐ Trusting relationships already exist with academic partners

The first four features are especially key where the community partner leads priority-setting. The inclusiveness of the priority-setting process will likely reflect the community partner's diversity, decision-making, and networks.

FEATURES OF INDIVIDUALS FROM THE COMMUNITY WHO MAKE GOOD PARTNERS:

- ☐ Collectively reflect the diversity of lived experience of using a service or a community (e.g. in disability research, this means selecting several individuals that span different types of disability — visual, hearing, mobility, psychosocial, cognitive)
- ☐ Are well-connected and informed across their community; have deep understanding of their community and the issues impacting it
- ☐ Willing to share their experiences and perspectives
- ☐ Can still be a voice for others and tell their stories; aren't fixated on their own problems
- ☐ Want to make a difference for others and the health system
- ☐ Are confident, assertive, vocal
- ☐ Have analytical skills or some research experience/ training
- ☐ Good team-work and interpersonal skills
- ☐ Good and conflict resolution skills

CASE EXAMPLE OF SELECTING PARTNERS: IDENTIFYING COMMUNITY PARTNERS WHERE NO EXISTING RELATIONSHIPS EXIST

The W-DARE project was an interdisciplinary partnership between researchers, health service providers, and disabled persons organisations (DPOs) that steered the engagement of women living with disability in Quezon City and Ligao City in the Philippines. The W-DARE project focused on improving the reproductive and sexual health of women living with disability in the Philippines. However, at the project's outset, researchers did not have established networks or relationships with many community partners in the Philippines. To identify community partners with whom to conduct the W-DARE project, researchers therefore undertook a systematic review of the different health service providers and disabled persons organisations, with a particular focus on those operating in Quezon City and Ligao City. The main criteria for selection were as follows:

1. Experience in working in the field (i.e. disability, gender, reproductive health)
2. Established networks in the community
3. Interest to join the project

According to a researcher: "The third criterion in particular was the main reason why we ended up with WOWLEAP since other disabled persons organisations we talked to were hesitant to be associated with a reproductive health project. Most disability organisations (and leaders) have close association with the Catholic Church. The WOWLEAP President agreed to join on behalf of WOWLEAP since she is open-minded, she trusted that we would not put her in a compromising position, and she recognised that reproductive health is also an important issue for women with disabilities. PARE (a disabled persons organisation) was selected because it had worked in both gender, disability, and reproductive health issues and likewise had networks it could tap into. Likhaan (a health service provider) was selected mainly because of their credibility in reproductive health and their instant full support to the project description that was circulated during the consultation I conducted."

2. FRAMING

How will you frame the priority-setting process to the community/academic partner(s) being approached?

Possible frames you might use:

OPEN SCOPE

Open scope: There is an open scope to set any topic and research questions, so this is an opportunity to identify and act on pressing academic or community research priorities.

ALIGNMENT

Alignment with existing academic, community organisation, or community priorities: The set topic (due to funding constraints) focuses on academic researchers' priorities, community organisation priorities or missions and/or community priorities. This is an opportunity to develop and test research questions on that topic, which will benefit the academic researchers or the community organisation and its community.

DUAL SCOPE

Dual scope: Although the priority-setting process's focus is already set by the funder, there is scope to build the invited academic or community partner's agenda into the process (see example below).

CAPACITY DEVELOPMENT

Helping partners learn new things and develop new capabilities: The partnership can build community organisation capacity to identify research topics and questions, and to undertake systematic data collection and analysis on its own.

It is possible to use frames 1, 2, or 3 in combination with frame 4. Draw on key informants' suggestions when selecting what frame(s) to use.

Selecting Partners Worksheet: Question for Reflection and Discussion

CASE EXAMPLE OF A DUAL SCOPE FRAMING

The Participation for Local Action (PLA) project in Karnataka, India was carried out by academic researchers in partnership with the Zilla Budakattu Girijana Abhivrudhhi Sangha, a district-level community development organisation representing the Soliga people. The Soliga are an Indigenous population who have lived in the BR Hills region of Southern Karnataka for centuries. There was not an open scope to focus the PLA project on what the district Sangha viewed as the Soliga people's priorities. The PLA project was focused on access to maternal and child health services, as per the awarded funding. So how could the community organisation's voice be reflected in setting the research project topic and research questions?

Before pitching the PLA project to the district Sangha, the academic researchers brainstormed how they could frame the partnership in ways that would benefit and be attractive to the community organisation. Importantly, they sought the advice of the project's co-investigator who was a member of the district Sangha. His suggestions guided how the principal investigator's proposal was framed:

> *"I think in our initial conversation itself, we mentioned that look this is our focus in the project we're trying to build. Now you might have some other focus, we will not interfere with, so we will be able to bring in resources in the form of supporting data collectors, now if you want to bring your own agenda on what these data collectors are likely to do when they visit and when they have these conversations, we do not restrict those things."*

When pitching the partnership, maternal and child health was described as an entry point; the priority-setting process could gather information about other health problems in the Soliga community too. This proposal was agreed to and actualised, with the district Sangha building their own agenda into the priority-setting process. Its leaders were interested in identifying what other problems and illnesses the Soliga people were experiencing and determining whether they were able to access government benefit schemes. Thus, problems within the Soliga community relating to maternal and child health-as well as wider health problems- were identified. After identifying these problems, priorities were set. The academic researchers agreed to take up other projects on those wider priorities to help the community after the PLA project finished.

TEAM ANSWER

NEXT STEPS

- Identify **Strategies and/or Actions to Take** to reach out to top candidates to pitch the partnership to them. Draw on key informants' knowledge and suggestions when determining how best to reach out to them.
- Once new partners have been found, then move to **Worksheet 2.**

STRATEGIES AND/OR ACTIONS TO TAKE
