# Supplementary material for: Sharing power in global health research: an ethical toolkit for designing priority-setting processes that meaningfully include communities
Source: Int J Equity Health. 2021 May 25;20:127. doi: 10.1186/s12939-021-01453-y (PMC8145852; doi:10.1186/s12939-021-01453-y)
Supplement: Supplementary file 3 — Additional file 3. ETHICAL TOOLKIT WORKSHEET 2 [file 12939_2021_1453_MOESM3_ESM.pdf]

ETHICAL TOOLKIT WORKSHEET 2

Deciding to Partner Worksheet: Questions for Reflection and Discussion

This worksheet should be completed by the research team collectively. Please first read the **Companion Document: Key Considerations in Worksheet 2** in order to understand how the questions in this worksheet relate to one another. Then complete Worksheet 2 as a team. To start, reflect on and discuss Question 1 collectively. Record your team's answer and read the Next Steps to take. Where the Next Steps ask you to identify Strategies and/or Actions to Take, do so as a team and record them before moving on to the next question in the worksheet.

1. BUILDING FOUNDATIONS

a. How will relationships between partners be built or made stronger?

Possible relationship building approaches you might use:

Informal interactions are key to forming personal connections between academic and community partners:

1. **Icebreakers**
2. **Tea and lunch breaks**
3. **Creative or shared activities** like crafting or harmonica lessons: Such activities are *"especially levelling"* because *"if you can learn to do something with someone where you're both equally unknowledgeable and unpractised, it creates a bond to start you off."* (community engagement practitioner, UK)
4. **Sensitivity training** for people without a disability to experience what it's like living with a disability: *"You do it and you understand. If you just listen sometimes, you will not feel it, but if you do it, you can."* (person living with disability, Philippines)

**TEAM ANSWER**

b. How will community partners be supported to participate in priority-setting?

Possible supports you might provide:

1. **Training** about grant writing and funding processes; ethics processes; and research processes, methods, and jargon
2. **Accommodating varying needs:** providing information in ways those engaged can understand and taking account of any disabilities (physical (mobility, vision, hearing), psychosocial, cognitive)
3. **Creating a safe space:** an atmosphere where people are encouraged to be critical and where people are comfortable with each other and with sharing their views and perspectives
4. **Pairing system:** linking an academic partner with a community partner
5. **Making community partners feel valued:** providing a comfortable venue with refreshments, remembering people's names and things about them, being friendly and welcoming, and making affirmative statements like "we really value you and feel you can make a valuable contribution".

TEAM ANSWER

NEXT STEPS

- Identify **Actions** to undertake selected relationship building activities and to provide the selected supports.
- Then move to Question 2.

STRATEGIES AND/OR ACTIONS TO TAKE

1. BARRIERS

What barriers still might exist to sharing power between partners?

Possible barriers that might exist at personal, relational, environmental, and normative levels:

- Lack of knowledge about research or the community
- Internalised assumption of knowing little relative to ‘expert’ academic researchers
- Feeling intimidated
- Lack of engagement experience
- Playing favourites
- Devaluing community knowledge
- Failure to accommodate varying needs (e.g. speaking different languages)
- Lack of funding
- Lack of time or capacity, competing commitments

TEAM ANSWER

NEXT STEPS

- Identify **Strategies and/or Actions to Take** to mitigate identified barriers. While it may only be within the research team's power to address personal and relational level barriers, do consider if anything can be done to mitigate environmental and normative barriers.
- Then move to Question 3.

STRATEGIES AND/OR ACTIONS TO TAKE

3. DECIDING TO PARTNER

Will foundations be sufficient and will barriers be adequately mitigated for the partnership to proceed?

TEAM ANSWER

NEXT STEPS

- If yes, move to **Worksheet 3**.
- If no, return to Questions 1 and 2 or consider collectively whether the partnership should move forward.
