# Supplementary material for: Sharing power in global health research: an ethical toolkit for designing priority-setting processes that meaningfully include communities
Source: Int J Equity Health. 2021 May 25;20:127. doi: 10.1186/s12939-021-01453-y (PMC8145852; doi:10.1186/s12939-021-01453-y)
Supplement: Supplementary file 4 — Additional file 4. ETHICAL TOOLKIT WORKSHEET 3 [file 12939_2021_1453_MOESM4_ESM.pdf]

ETHICAL TOOLKIT WORKSHEET 3

Deciding to Engage with the Wider Community Worksheet: Questions for Reflection and Discussion

This worksheet should be completed by the research team collectively. Please first read the **Companion Document: Key Considerations in Worksheet 3** in order to understand how the questions in this worksheet relate to one another. Then complete Worksheet 3 as a team. To start, reflect on and discuss Question 1 collectively. Record your team's answer and read the Next Steps to take. Where the Next Steps ask you to identify Strategies and/or Actions to Take, do so as a team and record them before moving on to the next question in the worksheet.

1. EXISTING PRIORITIES

a. Have the community's health research priorities, including those of the disadvantaged, less influential, lower status, and/or marginalised, already been voiced?

TEAM ANSWER

NEXT STEPS

- If your answer is **yes**, complete Question 1b as a research team.
- If your answer is **no**, skip Questions 1b and 1c and complete Question 2 as a research team.

b. What are the costs and benefits of doing a new priority-setting exercise?

TEAM ANSWER

COSTS	BENEFITS

NEXT STEPS

- Complete Question 1c as a research team.

c. Do the costs outweigh the benefits? Are the benefits small?

TEAM ANSWER

NEXT STEPS

- If your answer is yes, take a moment to step back and reflect on whether a new priority-setting exercise is necessary.
- If a new priority-setting exercise is NOT necessary, move to **Worksheet 4B**.
- If a new priority-setting exercise is necessary, complete Question 2 as a research team.

2. BUILDING FOUNDATIONS

a. How will relationships between the research team and the wider community be built or made stronger before priority-setting starts?

Possible relationship building approaches you might use:

Informal interactions are key to forming connections between researchers and communities:

1. **Community festivals** that open universities and research institutions to the public
2. **Film nights**
3. **Introductory/education sessions** with panels that combine academic researchers and people with lived experience who have been engaged in research
4. **Mailing lists** for engagement opportunities
5. **Time in the community:** Academic researchers spend time in the community outside of their research

Such activities largely have *"nothing to do with sitting around the table doing co-design with our researchers, but they also have everything to do with how we build our relationship with our community that leads to people wanting to come and sit at the table with us… we set up an expectation that what we really value is our difference of opinion… we are respectful of all voices and we wanna hear all voices."* (community engagement practitioner, UK)

Community engagement practitioners can also help the research team build links with their communities, assist them to come up with strategies and activities for how to do so, and support them to implement those strategies and activities.

TEAM ANSWER

b. How will community members be supported to participate in priority-setting?

Possible relationship building approaches you might use:

1. **Training** about grant writing and funding processes; ethics processes; research processes, methods, and jargon; and patient and public involvement roles in research
2. **Accommodating varying needs:** providing information in ways those engaged can understand and taking account of any disabilities (physical (mobility, vision, hearing), psychosocial, cognitive)
3. **Creating a safe space:** an atmosphere where people are encouraged to be critical and where the people are comfortable with each other and with sharing their views and perspectives
4. **Pairing system:** linking a researcher team member with a community member, linking a community member with little to no engagement experience with another community member with significant engagement experience
5. **Making community members feel valued:** providing a comfortable venue with refreshments, remembering people's names and things about them, being friendly and welcoming, and making affirmative statements like "we really value you and feel you can make a valuable contribution."

TEAM ANSWER

NEXT STEPS

- Identify **Actions** to undertake selected relationship building activities and to provide the selected supports.
- Then move to Question 3.

STRATEGIES AND/OR ACTIONS TO TAKE

3. BARRIERS

What barriers to meaningful engagement with the wider community might still exist?

Possible barriers that might exist at personal, relational, environmental, or normative levels:

- Lack of knowledge about research or the community
- Internalised assumption of knowing little relative to ‘expert’ academic researchers
- Feeling intimidated
- Lack of engagement experience
- Playing favourites
- Devaluing community knowledge
- Failure to accommodate varying needs (e.g. speaking different languages)
- Lack of funding
- Logistics (when and where engagement is undertaken)
- Lack of or inadequate compensation
- Previous negative or exploitative experiences when working with academic researchers
- Lack of diversity amongst community partner(s)’ members, including not encompassing those considered disadvantaged or marginalised in the community
- Community partner(s)’ networks do not reach those considered disadvantaged or marginalised in the community
- Community partner(s)’ decision-making processes are not participatory and do not rely on deliberative norms (e.g. equal opportunity to speak, question and answer, dissent, consensus expressed and discussed)

The latter three features are especially key where the community partner leads priority-setting; The inclusiveness of the priority-setting process will likely reflect its diversity, decision-making, and networks.

TEAM ANSWER

NEXT STEPS

- Identify **Strategies and/or Actions to Take** to mitigate identified barriers. While it may only be within the research team's power to address personal and relational level barriers, do consider if anything can be done to mitigate environmental and normative barriers.
- Then move to Question 4.

STRATEGIES AND/OR ACTIONS TO TAKE

4. DECIDING TO ENGAGE

Will foundations be sufficient and will barriers be adequately mitigated for the meaningful engagement with the community to proceed?

TEAM ANSWER

NEXT STEPS

- If **yes**, proceed to **Worksheet 4A**
- If **no**, return to Questions 2 and 3 and think about whether additional relationship building or supports are feasible or whether any remaining barriers can be further mitigated. If not, consider collectively whether engagement with the wider community should move forward.
