# Supplementary material for: Sharing power in global health research: an ethical toolkit for designing priority-setting processes that meaningfully include communities
Source: Int J Equity Health. 2021 May 25;20:127. doi: 10.1186/s12939-021-01453-y (PMC8145852; doi:10.1186/s12939-021-01453-y)
Supplement: Supplementary file 5 — Additional file 5. ETHICAL TOOLKIT WORKSHEET 4A [file 12939_2021_1453_MOESM5_ESM.pdf]

ETHICAL TOOLKIT WORKSHEET 4A

Designing Priority-setting Worksheet: Questions for Reflection and Discussion

This worksheet should be completed by the research team collectively. Please first read the **Companion Document: Key Considerations in Worksheet 4A** and the summary of the questions in this worksheet below.

After reading both, complete Worksheet 4A as a team. Before answering each question, read the Points to Consider (where provided). Then reflect on and discuss the question collectively. Record your team's answer and read the Next Steps to take. Where the Next Steps ask you to brainstorm Strategies and/or Actions to Take, do so as a team and record them before moving on to the next question in the worksheet.

If you find that you are unable to answer many of the questions in this worksheet, as a team, consider returning to Worksheet 3 and further reflecting on whether the foundations for meaningful engagement are present and/or how to further strengthen them.

SUMMARY OF WORKSHEET 4A QUESTIONS FOR REFLECTION AND DISCUSSION

1. LEADERSHIP
Who will lead the health research priority-setting process?

2. SCOPE
Will research topics be solicited relating to *all* health problems experienced by community members?

3. EMPOWERMENT
Will community partners be empowered as researchers during priority-setting?

Will community members' capacity to participate in research priority-setting be strengthened?

4. STAGE OF PARTICIPATION
What stage(s) of the priority-setting process do community partners want to be involved in?

What stage(s) of the priority-setting process will community members be involved in? Is this acceptable to them?*
*To ascertain once specific community members are invited to participate.

5. LEVEL OF PARTICIPATION
Will community members be involved as collaborators (decision-makers) and/or consultants? Is this acceptable to them?*
*To ascertain once specific community members are invited to participate.

6. DIVERSITY WITHIN THE COMMUNITY

a. Which community roles will you engage during priority-setting and for what reasons?
b. List which of the roles identified in Q6a correspond to greater or lesser influence and status within the community.
c. Who are considered disadvantaged, less influential, lower status, or marginalised within these roles?
d. Which of those groups or stakeholders in Q6c will you engage and for what reasons?
e. Is it fair to bring these community members into the same decision-making space?

7. REPRESENTATION
Which organisations or individuals will represent the roles listed in Q6a in priority-setting?

Do these representatives encompass those considered disadvantaged, less influential, lower status, and/or marginalised within each role, as identified in Question 6d?

8. MASS
Will the number of community partner staff and community members be greater or equal to academic partner staff in consultations and deliberations during priority-setting?

Will the number of representatives of lower status community roles (identified in Q6b) be sufficient at consultations and deliberations during priority-setting?

SUMMARY OF WORKSHEET 4B QUESTIONS FOR REFLECTION AND DISCUSSION

9.SPACE
Where will you hold the priority-setting process for your research project?

10. GROUND RULES
Will community partners and members be involved in developing and approving the ground rules for the priority-setting process? If not, what are your reasons?

What ground rules will you include to ensure stakeholders identified in Question 6d aren't silenced during priority-setting?

11. FACILITATION
Will you have a locally-based person facilitate consultations and deliberations during priority-setting? If not, what are your reasons?

How will the facilitation method/approach give participants an equal opportunity to speak at focus groups and deliberations during priority-setting?

How will the facilitation method/approach make community partners and members feel comfortable sharing relevant, personal stories about their community's health concerns during priority-setting?

12. LISTENING
How will the research team ensure community partners' and members' ideas are listened to during consultations and deliberations?

13. BEING HEARD
Will the voices of community members, especially those considered disadvantaged, less influential, lower status, and/or marginalised, have equal or greater weight than other participants' voices when setting research priorities? If not, what are your reasons?

14. RESOURCES AND COMPENSATION
How will community partners and communities be compensated for participating in priority-setting?

Will full information about the research project's budget be disclosed to community partners?

Will community partners have control over any project resources?

15. UNINTENDED HARMS
What harms do you think might result from the priority-setting process?

16. ACCOUNTABILITY
Will the research team act upon the final research topic and questions?

How will the final research topic and questions be fed back to field investigators and community members, including those considered disadvantaged, less influential, lower status, and/or marginalised, after priority-setting?

How are the research team and community members going to evaluate community members' engagement in the priority-setting process?

1. LEADERSHIP

Who will lead the health research priority-setting process?

TEAM ANSWER

NEXT STEPS

- Where those initiating priority-setting do not include locally-based academic researchers, community partners, or key informants:
  - » Discuss with locally-based academic researchers and community partners on the team whether they can take on this role,
  - » Discuss with community partners whether they have staff with the interest and capacity to be principal or co-investigators, and/or
  - » Look for locally-based or Indigenous academic researchers who are known to and trusted by the community to add as principal investigators or co-investigators.

- Develop a **Strategy** (if necessary) for approaching candidates about being principal or co-investigators and helping lead priority-setting.

- Proceed to Question 2.

STRATEGIES AND/OR ACTIONS TO TAKE

2. SCOPE

Will research topics be solicited relating to all health problems experienced by community members?

TEAM ANSWER

NEXT STEPS

- If your answer is **yes**, brainstorm how to explain to participants (at the start of priority-setting) that topics related to any or most health problems can be proposed during priority-setting and why that is the case. Then proceed to Question 3.
- If your answer is **no**, brainstorm how to explain to participants which health problems are off the table, and why, in order to avoid raising unrealistic expectations. Then proceed to Question 3.

STRATEGIES AND/OR ACTIONS TO TAKE

3. EMPOWERMENT

Will community partners be empowered as researchers during priority-setting?

Will community members' capacity to participate in research priority-setting be strengthened?

TEAM ANSWER

NEXT STEPS

- If your answer is **yes**, brainstorm **Strategies** for how the priority-setting process can build the knowledge, confidence, networks, and/or skills of community partners to identify research topics and questions on their own. Brainstorm **Strategies** for how the priority-setting process can build community members' capacity to participate in research priority-setting. Draw on community partners' and key informants' insights and recommendations when developing these strategies. Then proceed to Question 4.

- If your answer is **no** and the purpose of engagement is solely instrumental, it should be made transparent to those engaged from the wider community and justified to them. Brainstorm **Strategies** for how this will be done as a research team. Then proceed to Question 4.

STRATEGIES AND/OR ACTIONS TO TAKE

4. STAGE OF PARTICIPATION

What stage(s) of the priority-setting process do community partners want to be involved in?

What stage(s) of the priority-setting process will community members be involved in? Is this acceptable to them?*

*To ascertain once specific community members are invited to participate.

TEAM ANSWER

NEXT STEPS

- Consider whether your answers call for community partners and key informants from the community to be involved from the start of the process. If the answer is **yes**, brainstorm a **Strategy** to find out whether this entry point is acceptable to community members. Then proceed to Question 5.
- If the answer is **no**, discuss how it might be possible to include community partners and community members earlier in the priority-setting process. Brainstorm **Actions to Take** to do so and a **Strategy** to find out whether this entry point is acceptable to community members. Then proceed to Question 5.

STRATEGIES AND/OR ACTIONS TO TAKE

5. LEVEL OF PARTICIPATION

Will community members be involved as collaborators (decision-makers) and/or consultants? Is this acceptable to them?*

*To ascertain once specific community members are invited to participate.

Possible priority-setting mechanisms you might use:

A. Pure collaborative:
Hold a deliberative workshop or series of workshops with the research team and community members to solicit research topics, prioritise amongst them, and formulate research questions

B. Mix of collaborative and consultative:
1. Consult community members about the health problems they face in a single focus group discussion, in separate small focus group discussions, or individually through interviews
2. Research team analyses data at a deliberative analysis workshop to generate a list of research topics
3. Hold a deliberative workshop with the research team and community members to prioritise amongst the list of research topics and to formulate research questions

C. Pure consultative:
1. Consult community members about the health problems they face in a single focus group discussion, in separate small focus group discussions, or individually through interviews
2. Research team analyses data at a deliberative analysis workshop to generate a list of research topics
3. Research team prioritises amongst the list of research topics and formulates research questions at a deliberative workshop

Power is more evenly shared where community members participate in health research priority-setting as collaborators—Options A and B above.

Even so, pure consultative mechanisms can still share power with the community where locally-based academic researchers, community partners, and field investigators from the community participate in identifying research topics (data analysis), prioritising amongst them, and formulating research questions. Field investigators are individuals who collect and analyse data from the community via interviews and focus groups as part of consultative priority-setting processes.

TEAM ANSWER

NEXT STEPS

- If your answer to Question 5 is that community members will be involved as collaborators, consider whether and how research priorities can be set through a deliberative process with community members that yields a collective decision. Deliberative processes are governed by norms of having an equal opportunity to speak, to ask and answer questions, to dissent, and generate consensus. All participants can voice their ideas for research topics and explain why they favour them. Other participants can then ask them questions of clarification or contestation to which they can respond. All participants debate the pros and cons of various proposals. The final set of priorities is mutually agreed upon by all participants.

Brainstorm **Actions to Take** to run the deliberative workshop(s) below. Deliberative community engagement processes have been used to inform institutional ethics policies on biobanking and benefit sharing. Methods applied in these studies may be a rich resource to draw upon to inform health research priority-setting practice[1].

Also, brainstorm a **Strategy** to make sure this level of participation is acceptable to community participants. Then proceed to Question 6.

- If your answer to Question 5 is that community members will be involved as consultants, brainstorm how they will be consulted and how a ratification process involving community members, including those considered disadvantaged or marginalised, can be implemented for the final research topic and questions. Also, consider whether a diverse subset of community partner staff or community members can be selected and trained (as field investigators) to collect and analyse data from community members, and develop strategies for training them. Brainstorm **Actions to Take** to implement the consultation, ratification, and training processes below.

Also, brainstorm a **Strategy** to make sure this level of participation is acceptable to community participants. Then proceed to Question 6.

STRATEGIES AND/OR ACTIONS TO TAKE

[1]See: O'Doherty, K.C., Hawkins, A.K., & Burgess, M.M. (2012). Involving Citizens in the Ethics of Biobank Research: Informing Institutional Policy through Structured Public Deliberation. Social Science & Medicine. 75, 1604-1611; Marsh, V. et al. (2013). Consulting Communities on Feedback of Genetic Findings in International Health Research: Sharing Sickle Cell Disease and Carrier Information in Coastal Kenya. BMC Medical Ethics. 14, 41; Njue, M., Kombe, F., Mwalukore, S., Molyneux, S., & Marsh, V. (2014) What Are Fair Study Benefits in International. Health Research? Consulting Community Members in Kenya. PLoS ONE. 9(12), e113112. doi:10.1371/journal.pone. 0113112

STRATEGIES AND/OR ACTIONS TO TAKE CONTINUED

6. DIVERSITY WITHIN THE COMMUNITY

6a. Which community roles will you engage during priority-setting and for what reasons?

6b. List which of the roles identified in Q6a correspond to greater or lesser influence and status within the community.

6c. Who are considered disadvantaged, less influential, lower status, or marginalised within these roles?

6d. Which of those groups or stakeholders in Q6c will you engage and for what reasons?

6e. Is it fair to bring these community members into the same decision-making space?

Points to consider:
1. Can or does the research team need to undertake stakeholder mapping with community partners, and/or key informants in order to identify what roles are present in the community?

2. Which of those roles are relevant to include as participants? For example, advancing values of equity and social justice speak to two main reasons for selecting particular community roles to involve in priority-setting: because they either have pertinent knowledge of the health needs of those considered disadvantaged or marginalised, or because they have the power to change policies and practices that affect those individuals or groups' health. In a recent priority-setting process with women living with disability, relevant roles included: women living with disability, carers and families, and health service providers.

3. Can the research team develop a definition of what constitutes 'disadvantage', 'less influential', 'lower status', and/or 'marginalisation' in the community with community partners, field investigators, and/or key informants and use it to identify lower status roles and those who are considered disadvantaged, less influential, lower status, and/or marginalised within the relevant roles?

4. When is it unfair to bring certain community members into the same decision-making space?

In certain contexts, pure collaborative or mixed collaborative and consultative priority-setting mechanisms may not be appropriate. It may not be fair to bring certain community members into the same deliberative decision-making space. Three examples of such circumstances are provided:

- Oppressor situation- Certain community members are responsible for oppressing other community members. The oppressors and/or the community members they oppress will require separate consultations.
- Power disparities situation- Power disparities are too large to mitigate sufficiently between certain community members. Those community members who are unlikely to raise their voice in the presence of others may require separate consultations.
- Missing foundations situation- Respect and trust is lacking between community members. Certain community members may treat others in a very disrespectful manner when in the same space. Without trust, certain community members may not feel comfortable sharing their ideas and perspectives in front of others. To avoid harms of disrespect and to ensure the voices of all participants are heard may require performing separate consultations.

Where it is not fair to bring community members into the same decision-making space, pure consultative mechanisms may be the most appropriate way to access their voices. Power can still be shared with the community using such mechanisms, particularly where community members are consulted by field investigators from the community. And where data from consultations is analysed by research team members that include locally-based academic researchers, community partners, and field investigators from the community.

TEAM ANSWER

6b.

Greatest ⟷ Least

TEAM ANSWER

6c.

Greatest ⟷ Least

7. REPRESENTATION

Which organisations or individuals will represent the roles listed in Q6a in priority-setting?

Do these representatives encompass those considered disadvantaged, less influential, lower status, and/or marginalised within each role, as identified in Question 6d?

Points to consider:

1. Does it make sense to ask community leaders, field investigators, or key informants to select individuals or organisations to represent the identified roles? If yes, consider giving them some criteria that you are hoping representatives will meet in order to avoid selection biases.
2. Do you have evidence that selected organisations' memberships reflect the diversity of the role they represent and are regularly consulted about their needs and priorities? Returning to the example of a priority-setting process with women living with disability, selected disabled persons organisations spanned the diversity of types of disability: mobility, visual, hearing, psychosocial and cognitive.
3. Where individuals will represent a role, do they collectively reflect its diversity and share lived experience with those they are representing? Returning to the same example, selected individuals with lived experience spanned the diversity of types of disability: mobility, visual, hearing, psychosocial and cognitive.
4. Do any of the selected representatives have substantial financial conflicts of interest that you think will bias their identification of research priorities? For example, patient organisations funded by pharmaceutical companies.

TEAM ANSWER

NEXT STEPS

- As a research team, develop **Strategies** to recruit identified representatives. One option could be to ask community partners, field investigators, community leaders or key informants to recruit them, particularly those who may be hard to reach.
- Move to Question 8.

STRATEGIES AND/OR ACTIONS TO TAKE

8. MASS

Will the number of community partner staff and community members be greater or equal to academic partner staff in consultations and deliberations during priority-setting?

Will the number of representatives of lower status community roles (identified in Q6b) be sufficient at consultations and deliberations during priority-setting?

TEAM ANSWER

NEXT STEPS

- If your answers are **yes**, proceed to Question 9.
- If either answer is **no**, brainstorm with community partners, field investigators, and key informants whether there are additional organisations or individuals who could represent community members and/or lower status roles. Develop Strategies to recruit them to participate in priority-setting. Then move to Question 9.

STRATEGIES AND/OR ACTIONS TO TAKE

9. SPACE

Where will you hold the priority-setting process for your research project?

Points to consider:

- Is the space you have selected physically accessible and safe for those considered disadvantaged or marginalised within the community? Draw on community partners', field investigators', and key informants' knowledge to make this assessment.
- What norms are associated with the space? Do these norms silence those considered disadvantaged or marginalised? Draw on community partners, field investigators, and key informants' knowledge to make this assessment.

TEAM ANSWER

NEXT STEPS

- If the chosen space is physically accessible, safe, and not imbued with norms that will silence those considered disadvantaged or marginalised, develop a **Strategy** for gaining permission to use it for your priority-setting process. Then proceed to Question 10.
- If the space does not meet those criteria, brainstorm other possible locations that do and develop a **Strategy** for gaining permission to use one of them. Then proceed to Question 10.

STRATEGIES AND/OR ACTIONS TO TAKE

10. GROUND RULES

Will community partners and members be involved in developing and approving the ground rules for the priority-setting process? If not, what are your reasons?

What ground rules will you include to ensure stakeholders identified in Question 6d aren't silenced during priority-setting?

TEAM ANSWER

NEXT STEPS

- As a research team, develop a **Strategy** to set ground rules for the priority-setting process. To help involve community members, including those considered disadvantaged or marginalised, in selecting ground rules, the following steps are suggested:
  - Establish an initial list of ground rules and circulate it to participants, giving them time to comment. Then revise the list and get it approved by all participants before priority-setting starts.
  - When developing the initial list of ground rules, ask community partners, field investigators, and participants what rules are essential to ensure community members, including those considered disadvantaged or marginalised, have an equal opportunity to share their views during priority-setting.

- Proceed to Question 11.

STRATEGIES AND/OR ACTIONS TO TAKE

11. FACILITATION

Will you have a locally-based person facilitate consultations and deliberations during priority-setting? If not, what are your reasons?

How will the facilitation method/approach give participants an equal opportunity to speak at focus groups and deliberations during priority-setting?

How will the facilitation method/approach make community partners and members feel comfortable sharing relevant, personal stories about their community's health concerns during priority-setting?

Points to consider:

- Would a "stepped" approach be appropriate? In a "stepped" approach, small groups with some degree of homogeneity or similar characteristics deliberate first before everyone (or representatives of each small group) comes together as a large group. Such an approach helps reduce the impact of power disparities between groups by giving those who might otherwise be silenced a safe space to express themselves and to reflect on their ideas before having to present them to a wider audience.

- Will field investigators (i.e. interviewers, data collectors) share lived experience with interviewees? Such an approach facilitates interviewees feeling more comfortable sharing their stories and views.

- Can interviews, focus groups, and/or deliberations incorporate the use of local languages and ways of speaking like storytelling, pictures, dramas, and songs? Such an approach may promote interviewees having an equal opportunity to speak and make them feel more comfortable sharing their stories and views.

TEAM ANSWER

Designing Priority-setting Worksheet: Questions for Reflection and Discussion

TEAM ANSWER

NEXT STEPS

- As a research team, brainstorm who from the community or community partner has a strong understanding of community members' relationships and hierarchies, speaks local languages, and is acceptable and trusted by participants and, therefore, would make a good facilitator. Develop a **Strategy** with community partners to approach the top candidates.

- Then move to Question 12.

STRATEGIES AND/OR ACTIONS TO TAKE

12. LISTENING

How will the research team ensure community partners' and members' ideas are listened to during consultations and deliberations?

TEAM ANSWER

NEXT STEPS

- As a research team, brainstorm who from the community partner or within the community could be asked to document the priority-setting process. Develop a **Strategy** to approach the top candidates.
- As a research team, brainstorm how community members, including those considered disadvantaged or marginalised, can be given a fair opportunity to review the documentation of the priority-setting process.
- Move to Question 13.

STRATEGIES AND/OR ACTIONS TO TAKE

13. BEING HEARD

Will the voices of community members, especially those considered disadvantaged, less influential, lower status, and/or marginalised, have equal or greater weight than other participants' voices when setting research priorities? If not, what are your reasons?

TEAM ANSWER

NEXT STEPS

- If your answer is **yes**, brainstorm what might comprise reasonable rationales for giving certain community members' voices greater weight than others in health research priority-setting. Many voices, including opposing ones, will be raised in interviews, focus groups, and deliberations and this will create a dilemma of whose voices to use and not use. In practice, rationales that have been used include: amplifying unheard voices, amplifying voices that will create change, amplifying voices based on the values they further, relevance of the information provided, expertise of the community member providing the information, and avoiding reinforcing negative stereotypes of certain groups. Next, move to Question 14.

- If your answer is **no**, plan to be transparent and brainstorm how you will clarify this decision and the reasons behind it to participants. Then move to Question 14.

STRATEGIES AND/OR ACTIONS TO TAKE

14. RESOURCES AND COMPENSATION

How will community partners and communities be compensated for participating in priority-setting?

Will full information about the research project's budget be disclosed to community partners?

Will community partners have control over any project resources?

TEAM ANSWER

NEXT STEPS

- As a research team, brainstorm **Actions to Take** to organise employment contracts for community partners (as needed) and field investigators and consider whether fixed term contracts are possible. Investigate how community members would like to be compensated for their time and transport and brainstorm **Actions to Take** to organise it.
- Set a time to go through budgetary aspects of the project with community partners. This could be in relation to a set budget if funding has already been awarded or the budget requirements for the specific grant scheme to which the research team is applying.
- Move to Question 15.

STRATEGIES AND/OR ACTIONS TO TAKE

15. UNINTENDED HARMS

What harms do you think might result from the priority-setting process?

TEAM ANSWER

NEXT STEPS

- As a research team, develop **Strategies** to avoid or minimise the harms you have identified. Draw on community partners', field investigators', and key informants' insights and recommendations when doing so.
- Move to Question 16.

STRATEGIES AND/OR ACTIONS TO TAKE

16. ACCOUNTABILITY

Will the research team act upon the final research topic and questions?

How will the final research topic and questions be fed back to field investigators and community members, including those considered disadvantaged, less influential, lower status, and/or marginalised, after priority-setting?

How are the research team and community members going to evaluate community members' engagement in the priority-setting process?

TEAM ANSWER

NEXT STEPS

- As a research team, discuss what you will do with any comments you receive from field investigators and community members on the final research topic and questions.
- As a research team, identify **Actions to Take** to implement your plan for evaluating community members' participation in the priority-setting process.

STRATEGIES AND/OR ACTIONS TO TAKE

FINAL STEP: Based on your team's answers in this worksheet, develop a final priority-setting plan for your research project.
