# Supplementary material for: Sharing power in global health research: an ethical toolkit for designing priority-setting processes that meaningfully include communities
Source: Int J Equity Health. 2021 May 25;20:127. doi: 10.1186/s12939-021-01453-y (PMC8145852; doi:10.1186/s12939-021-01453-y)
Supplement: Supplementary file 6 — Additional file 6. ETHICAL TOOLKIT WORKSHEET 4B [file 12939_2021_1453_MOESM6_ESM.pdf]

ETHICAL TOOLKIT WORKSHEET 4B

Designing Priority-setting Worksheet: Questions for Reflection and Discussion

This worksheet should be completed by the research team collectively. Please first read the **Companion Document: Key Considerations in Worksheet 4B** and the summary of the questions in this worksheet below.

After reading both, complete Worksheet 4B as a team. Before answering each question, read the Points to Consider (where provided). Then reflect on and discuss the question collectively. Record your team's answer and read the Next Steps to take. Where the Next Steps ask you to brainstorm Strategies and/or Actions to Take, do so as a team and record them before moving on to the next question in the worksheet.

If you find that you are unable to answer many of the questions in this worksheet, as a team, consider returning to Worksheet 2 and further reflecting on whether the foundations for partnership are present and/or how to further strengthen them.

SUMMARY OF WORKSHEET 4B QUESTIONS FOR REFLECTION AND DISCUSSION

1. LEADERSHIP
Who will lead the health research priority-setting process?

2. EMPOWERMENT
Will community partners be empowered as researchers during priority-setting?

3. STAGE OF PARTICIPATION
What stage(s) of the priority-setting process do community partners want to be involved in? Will they be involved from the start of the process?

4. LEVEL OF PARTICIPATION
Do community partners want to be involved as collaborators (decision-makers) or consultants?

5. SPACE
Where will you hold the priority-setting process for your research project?

6. MASS
Will the number of community partner staff and academic partner staff participating in priority-setting be similar? Will similar numbers of different community partners participate? If not, what are the reasons?

7. GROUND RULES
Will community partners be involved in developing and approving the ground rules for the priority-setting process? If not, what are your reasons?

What ground rules will be included to ensure community partners have an equal opportunity to speak relative to academic partners, are listened to, and are heard during priority-setting?

8. FACILITATION
Will you have a locally-based person facilitate consultations and deliberations during priority-setting? If not, what are your reasons?

How will the facilitation method/approach give participants an equal opportunity to speak during priority-setting?

How will the facilitation method/approach make community partners feel comfortable sharing relevant, personal stories about their community's health concerns and their ideas for research priorities during priority-setting?

9. LISTENING
How will the research team ensure community partners' ideas are listened to during the priority-setting process?

SUMMARY OF WORKSHEET 4B QUESTIONS FOR REFLECTION AND DISCUSSION

10. HEARD
Will community partners’ voices have equal or greater weight in the priority-setting process relative to academic partners’ voices?

11. RESOURCES AND COMPENSATION
How will community partners be compensated for participating in priority-setting?

Will full information about the research project’s budget be disclosed to community partners?

Will community partners have control over any project resources?

12. ACCOUNTABILITY

Has the wider community endorsed the selected research topic and questions?

Will the research team act upon the final research topic and questions?

How are academic and community partners going to evaluate research partners’ participation in the priority-setting process?

1. LEADERSHIP

Who will lead the health research priority-setting process?

TEAM ANSWER

NEXT STEPS

- Where those initiating priority-setting do not include locally-based academic researchers, community partners, or key informants:
  - » Discuss with locally-based academic researchers and community partners on the team whether they can take on this role
  - » Discuss with community partners whether they have staff with the interest and capacity to be principal or co-investigators, and/or
  - » Look for locally-based or Indigenous academic researchers who are known to and trusted by the community to add as principal investigators or co-investigators.

- Develop a **Strategy** (if necessary) for approaching candidates about being principal or co-investigators and helping lead priority-setting.

- Proceed to Question 2.

STRATEGIES AND/OR ACTIONS TO TAKE

2. EMPOWERMENT

Will community partners be empowered as researchers during priority-setting?

TEAM ANSWER

NEXT STEPS

- If your answer is **yes**, brainstorm Strategies for how the priority-setting process can build the knowledge, confidence, networks, and/or skills of community partners to identify research topics and questions on their own. Then proceed to Question 3.
- If your answer is **no** and the purpose of engagement is solely instrumental, it should be made transparent to community partners and justified to them. Discuss this matter as a research team. Then proceed to Question 3.

STRATEGIES AND/OR ACTIONS TO TAKE

3. STAGE OF PARTICIPATION

What stage(s) of the priority-setting process do community partners want to be involved in? Will they be involved from the start of the process?

TEAM ANSWER

NEXT STEPS

- If your answer is community partners will be involved from the start of priority-setting (i.e. conceptualising the process), then proceed to Question 4.
- If not, discuss how it might be possible to include community partners earlier in the priority-setting process. Where existing community partners do not want to be part of the early stages of priority-setting, discuss the possibility of and/ or need to involve additional community partners or key informants. Brainstorm **Actions to Take** to approach them if you decide doing so is needed. Then proceed to Question 4.

STRATEGIES AND/OR ACTIONS TO TAKE

4. LEVEL OF PARTICIPATION

Do community partners want to be involved as collaborators (decision-makers) or consultants?

TEAM ANSWER

NEXT STEPS

- If community partners want to be involved as collaborators, consider whether and how the research topic and question can be set through a deliberative workshop or series of workshops that yield a collective decision. Deliberative processes are governed by norms of having an equal opportunity to speak, to ask and answers questions, to dissent, and generate consensus. All participants can voice their ideas for research priorities and explain why they favour them. Other participants can then ask them questions of clarification or contestation to which they can respond. All participants debate the pros and cons of various proposals. The final research topic and questions are mutually agreed upon by all participants.

  Brainstorm **Actions to Take** to run the deliberative workshop(s) below. Deliberative community engagement processes have been used to inform institutional ethics policies on biobanking and benefit sharing. Methods applied in these studies may be a rich resource to draw upon to inform health research priority-setting practice[1].

  Then proceed to Question 5.

- If community partners want to be involved as consultants, discuss how they will be consulted and whether a process where they ratify the identified research topic and questions can be implemented. Brainstorm **Actions to Take** to implement the consultation and ratification processes below. Then proceed to Question 5.

STRATEGIES AND/OR ACTIONS TO TAKE

[1]See: O'Doherty, K.C., Hawkins, A.K., & Burgess, M.M. (2012). Involving Citizens in the Ethics of Biobank Research: Informing Institutional Policy through Structured Public Deliberation. Social Science & Medicine. 75, 1604-1611; Marsh, V. et al. (2013). Consulting Communities on Feedback of Genetic Findings in International Health Research: Sharing Sickle Cell Disease and Carrier Information in Coastal Kenya. BMC Medical Ethics. 14, 41; Njue, M., Kombe, F., Mwalukore, S., Molyneux, S., & Marsh, V. (2014) What Are Fair Study Benefits in International. Health Research? Consulting Community Members in Kenya. PLoS ONE. 9(12), e113112. doi:10.1371/journal.pone. 0113112

5. SPACE

Where will you hold the priority-setting process for your research project?

Points to consider:

- Is the space you have selected physically accessible for community partners?
- What norms are associated with the space? Are they likely to make community partners feel less comfortable sharing their ideas and experiences?

TEAM ANSWER

NEXT STEPS

- If the chosen space is physically accessible and not imbued with norms that will silence community partners, develop a **Strategy** for gaining permission to use it for your priority-setting process. Then proceed to Question 6.
- If the space does not meet those criteria, brainstorm other possible locations that do and develop a **Strategy** for gaining permission to use one of them. Then proceed to Question 6.

STRATEGIES AND/OR ACTIONS TO TAKE

6. MASS

Will the number of community partner staff and academic partner staff participating in priority-setting be similar? Will similar numbers of different community partners participate? If not, what are the reasons?

TEAM ANSWER

NEXT STEPS

- If your answer to Question 6 is **yes**, proceed to Question 7.
- If your answer to Question 6 is **no**, consider whether it is possible to involve more staff from those partners with fewer numbers. Brainstorm **Strategies** to recruit them to participate in priority-setting. Where involving more staff is not possible, discuss how to reduce large disparities in numbers between academic and community partners in priority-setting. Then proceed to Question 7.

STRATEGIES AND/OR ACTIONS TO TAKE

7. GROUND RULES

Will community partners be involved in developing and approving the ground rules for the priority-setting process? If not, what are your reasons?

What ground rules will be included to ensure community partners have an equal opportunity to speak relative to academic partners, are listened to, and are heard during priority-setting?

TEAM ANSWER

NEXT STEPS

- As a research team, develop a **Strategy** to set ground rules for the priority-setting process. The following steps are suggested:
  - » Establish an initial list of ground rules and circulate it to all research partners, giving them time to comment. Then revise the list and get it approved by all partners before priority-setting starts.
  - » When obtaining comments on the initial list of ground rules, ask partners to add additional ground rules if those proposed are insufficient to ensure community partners have an equal opportunity to speak, be listened to, and be heard relative to academic partners during priority-setting.

- Then move to Question 8.

STRATEGIES AND/OR ACTIONS TO TAKE

8. FACILITATION

Will you have a locally-based person facilitate consultations and deliberations during priority-setting? If not, what are your reasons?

How will the facilitation method/approach give participants an equal opportunity to speak during priority-setting?

How will the facilitation method/approach make community partners feel comfortable sharing relevant, personal stories about their community's health concerns and their ideas for research priorities during priority-setting?

Points to consider:

- Would a "stepped" approach be appropriate? In a "stepped" approach, small groups with some degree of homogeneity or similar characteristics deliberate first before everyone (or representatives of each small group) comes together as a large group. Such an approach helps reduce the impact of power disparities between groups by giving those who might otherwise be silenced a safe space to express themselves and to reflect on their ideas before having to present them to a wider audience.

- What personal, cultural, environmental, or normative factors might make research team members less likely to share their views and ideas?

TEAM ANSWER

NEXT STEPS

- As a research team, brainstorm who from the community or community partner has a strong understanding of research partners' relationships and hierarchies, speaks local languages, and is acceptable and trusted by research partners and, therefore, would make a good facilitator. Develop a **Strategy** with community partners to approach the top candidates.

- Then move to Question 9.

STRATEGIES AND/OR ACTIONS TO TAKE

9. LISTENING

How will the research team ensure community partners' ideas are listened to during the priority-setting process?

TEAM ANSWER

NEXT STEPS

- As a research team, brainstorm who within the research team could document the priority-setting process. Develop a **Strategy** to approach the top candidates.
- As a research team, brainstorm how all partners can be given a fair opportunity to review the documentation of the priority-setting process.
- Move to Question 10.

STRATEGIES AND/OR ACTIONS TO TAKE

10. BEING HEARD

Will community partners' voices have equal or greater weight in the priority-setting process relative to academic partners' voices?

TEAM ANSWER

NEXT STEPS

- If your answer is **yes**, brainstorm how consultations or deliberations will be structured to achieve that. Then move to Question 11.
- If your answer is **no**, discuss what the justification is and whether it is acceptable to community partners. Then move to Question 11.

STRATEGIES AND/OR ACTIONS TO TAKE

11. RESOURCES AND COMPENSATION

How will community partners be compensated for participating in priority-setting?

Will full information about the research project's budget be disclosed to community partners?

Will community partners have control over any project resources?

TEAM ANSWER

NEXT STEPS

- As a research team, brainstorm **Actions to Take** to organise employment contracts for community partners and consider whether fixed term contracts are possible. Then move to Question 12.

- Set a time to go through budgetary aspects of the project with community partners. This could be in relation to a set budget if funding has already been awarded or the budget requirements for the specific grant scheme to which the research team is applying. Then move to Question 12.

STRATEGIES AND/OR ACTIONS TO TAKE

12. ACCOUNTABILITY

Has the wider community endorsed the selected research topic and questions?

Will the research team act upon the final research topic and questions?

How are academic and community partners going to evaluate research partners' participation in the priority-setting process?

TEAM ANSWER

NEXT STEPS

- As a research team, brainstorm a **Strategy** for how you will obtain community approval and/or feedback on the research topic and questions selected by the research team. Discuss what you will do with any comments you receive from community members on the final research priorities.

- As a research team, identify **Actions to Take** to implement your plan for evaluating research partners' participation in the priority-setting process.

STRATEGIES AND/OR ACTIONS TO TAKE

FINAL STEP: Based on your team's answers in this worksheet, develop a final priority-setting plan for your research project.
